# Phytochemical profiling, metabolomics, and molecular docking studies of *Atriplex halimus* aerial parts revealing potential insecticidal activity against the malaria vector *Anopheles pharoensis*

**DOI:** 10.1038/s41598-026-52695-1

**Published:** 2026-05-21

**Authors:** Esraa A. Elhawary, Hassan O. Waheeb, Abeer H. A. Abdelhafiz, Amr A. El-Waseif, Ahmed Z.I. Shehata

**Affiliations:** 1 Department of Pharmacognosy, Faculty of Pharmacy, Ain Shams University, Cairo, 11566 Egypt; 2 Department of Zoology, Faculty of Science (Boys), Al-Azhar University, Cairo, 11651 Egypt; 3 Department of Pharmaceutical Chemistry, Faculty of pharmacy, Ain Shams University, Cairo, 11566 Egypt; 4 Botany and Microbiology Dept, Faculty of Science (Boys), Al-Azhar University, Cairo, 11651 Egypt

**Keywords:** UPLC/MS analysis, Phytochemical profiling, *Atriplex halimus*, Antimicrobial activity, Mosquito repellency, *Anopheles pharoensis*, Biochemistry, Biotechnology, Chemical biology, Drug discovery, Microbiology, Plant sciences

## Abstract

*Atriplex halimus* is traditionally used in folk medicine for the treatment of skin infections, inflammation, and parasitic disorders. The aerial parts of *A. halimus* were extracted with 70% methanol and fractionated into dichloromethane (DCM), ethyl acetate (EtOAc), *n*-butanol (BuOH), and aqueous fractions. Comprehensive UPLC/MS profiling identified seventy-eight secondary metabolites, predominantly flavonoids and their derivatives, followed by triterpenoids and minor constituents. The antimicrobial activities of the extract and its fractions were assessed against *Staphylococcus aureus* and *Escherichia coli*. The methanol extract (12.0 and 30.0 mm) and the EtOAc fraction (14.0 and 22.0 mm) exhibited the strongest inhibitory effects. Repellent bioassays against *Anopheles pharoensis* starved females demonstrated solvent- and dose-dependent variations, with the DCM fraction at 6.67 mg/cm² achieving the highest repellency (81.92%). Molecular docking studies were further conducted to elucidate the interactions responsible for the observed antimicrobial and insecticidal activities. In conclusion, these findings highlight *A. halimus* aerial parts as a rich source of bioactive metabolites with dual antimicrobial and mosquito-repellent potential, supporting their application in natural product-based vector control strategies.

**Supplementary Information:**

The online version contains supplementary material available at 10.1038/s41598-026-52695-1.

## Introduction

Genus *Atriplex* comprises about 250 plant species and the genus has many common names viz. saltbush and orache. *Atriplex halimus* family Chenopodiaceae is a halophytic perennial shrub that thrives in arid and semi-arid environments. Its tolerance to high salinity and drought makes it an ideal species for landscaping in arid and salt-affected areas, where it provides valuable forage for livestock. *A. halimus* can grow from Europe to Northern Africa, Western Asia and Iraq as well as the Arabian Peninsula^1^. The plant leaf is traditionally used by Arab population in order to heal from rheumatism, diabetes and heart conditions. The plant is reported also for breast cancer and as laxative. *A. crossifolia* play a role in jaundice, while *A. hortensis* leaves are excellent as diuretic and purgative agent. Moreover, *A. sagittata* is listed for diabetes. The plant itself is edible due to high nutrients content especially protein and several other *Atriplex* species were listed in the same category including *A. hortensis*, *A. partulacoides* (act as functional food) and *A. sagittata* being used as leafy vegetables. *A. halimus* is reported to be rich in many classes of phytoconstituents such as tannins, flavonoids, saponins, and alkaloids. These compounds are potentially useful for the prevention against several human diseases and food preservation^2^.

Literature has reported many biological roles for *Atriplex* including antioxidant, anticholinesterase, antibacterial, antifungal, antiparasitic activity in vitro while many in vivo studies reported antidiabetic, hepatoprotective and a nephroprotective effects. The activities are usually tied to the presence of different phytochemical classes such as triterpenes, sterols and phytoecdysteroids, saponins and phenolic compounds. Phytochemical studies reported different types of flavonoids in many species of the genus *Atriplex viz.* the famous aglycones, quercetin and kaempferol together with their glycosides^3^.

This study aimed to investigate the phytochemical profile of *Atriplex halimus* aerial parts and its polarity-based fractions using UPLC/MS, and to compare their profiles through clustered heat map analysis. Additionally, the potential insecticidal activity of the extracts was evaluated. The most abundant bioactive compounds were further examined through molecular docking studies to gain insights into the observed insecticidal activity, as well as their antimicrobial effects against various microorganisms.

## Results

### Phytochemical constituents UPLC/ESI/MS tentatively identified from the 70% methanol extract and fractions of *Atriplex halimus* aerial parts

**Fig. 1:**
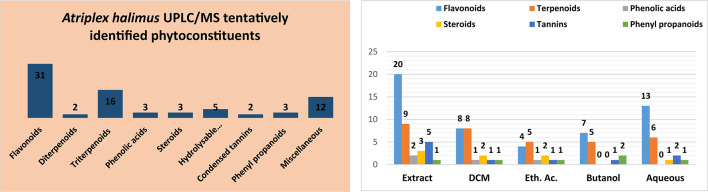
Bar charts showing (**A**) the different phytochemical classes of compounds (**B**) the main phytochemical components detected from the crude extract and fractions of *A. halimus*.

Seventy-eight compounds were tentatively, belonging to different classes of phytoconstituents, were putatively identified from the 70% methanol extract of the aerial parts of *Atriplex halimus* and its polar and non-polar fractions as presented in Suppl. Table 1. The majority of the identified compounds were from the flavonoids and their derivatives followed by terpenoids and many other classes as illustrated in Fig. 1A,B. The BPI chromatograms in ESI negative and positive ion modes for the extract and fractions were illustrated in Suppl. Figure S1 and Suppl. Figure S2. All identified compounds were tentatively assigned based on low-resolution UPLC–MS data, supported by literature comparison.

### Flavonoids

Flavonoids represent the main identified class of compounds from *Atriplex halimus* extract and fractions showing thirty-one compounds which is nearly half of the total number of identified compounds. They were heavily found in the methanol extract together with the two polar fractions (ethyl acetate and butanol) as shown in Suppl. Table 1.

Flavonoids and their derivatives were the most provisionally assigned class of compounds from the methanol extract of *A. halimus* and its fractions. A deprotonated molecular ion peak was detected at [M-H]^−^ *m/z* 273 and was assigned to *tetra*-hydroxy-flavan. A bi-flavonoid derivative showed it deprotonated peak at *m/z* 591 in ESI positive ion mode and was defined as manniflavanone (bi-flavanone), this peak showed characteristic fragments at *m/z* 391, 375, 299 and 135 due to flavanone ring cleavage, CO loss, cleavage of the monomeric flavanone ring and A-ring fragmentation, respectively. A molecular ion peak was shown at *m/z* 311 in ESI positive ion mode for a fragment of luteolin. Similarly, another luteolin fragment was detected at *m/z* 455 also in the ESI positive ion mode for a fragment of luteolin-hexoside. A third luteolin derivative had its deprotonated parent peak at *m/z* 737 in ESI –ve mode and a fragment of luteolin-*C*-hexoside-deoxyhexoside was also detected at *m/z* 473 in positive mode. In addition to that, different quercetin derivatives were recorded such as the famous hexoside named quercetin-hexoside which presented a parent peak at [M-H]^−^ *m/z* 463 with a key fragment at *m/z* 301 for the loss of the quercetin aglycone itself. Moreover, a parent peak was detected in the –ve ion mode at *m/z* 595 for another quercetin derivative named quercetin pentosyl hexoside (Suppl. Table 1).

A deprotonated peak was shown at *m/z* 505 in ESI –ve mode and at *m/z* 553 in ESI positive ion mode for the quercetin derivative, quercetin-acetyl-hexoside where the increment in the *m/z* ratio in the ESI positive ion mode was attributed to the presence of acetonitrile adduct. Similar group of kaempferol derivatives were identified at different *m/z* values namely; kaempferol-pentose-hexuronic acid which showed a parent peak at *m/z* 617 in ESI negative ion mode (3.39% of the aqueous fraction)^4^.

A deprotonated peak was presented at [M-H]^−^ *m/z* 593 and [M + H+CF_2_]^+^ *m/z* 645 and was assigned to kaempferol-deoxyhexosyl hexoside (5.41%, ethyl acetate fraction). An acetylated derivative of kaempferol showed a deprotonated peak at *m/z* 635 in ESI positive ion mode and was identified as acetylated kaempferol deoxyhexosyl hexoside. Similarly, the presence of a parent peak at *m/z* 577 in –ve mode was shown to be kaempferol-*di*-deoxyhexoside also known as kaempferitrin. Two chrysoeriol derivatives were characterized from the butanol and aqueous fractions of *A. halimus* where a deprotonated peak in ESI positive ion mode at *m/z* 625 was assigned to chrysoeriol-hexoside-hexoside (16.93%, aqueous fraction) while a parent peak appearing at [M-H]^−^ *m/z* 607 and [M + H]^+^ *m/z* 609 and characteristic fragment at *m/z* 481 due to loss of 126 Da of the deoxysugar thus it was likely identified as chrysoeriol-deoxyhexoside-hexoside (5.09%, aqueous fraction).

In addition to that, two scutellarein derivatives were recorded where a deprotonated peak was detected at *m/z* 564 in ESI –ve mode for scutellarein-pentosyl-pentoside together with a molecular ion peak at *m/z* 534 also in the negative mode and was assigned to scutellarein-(malonyl-hexoside) which is also known as scutellarein-(malonyl-hexoside)].

Other flavonoids and their derivatives were also detected such as for compound 21 which showed a deprotonated peak at *m/z* 329 in negative mode and was provisionally named tricin aglycone (E) (5.65%, 4.56% and 4.19% from the DCM, ethyl acetate and butanol fractions, respectively). Moreover, compound 60 presented a parent peak at [M + H]^+^ *m/z* 639 and was defined as tricin-deoxyhexosyl- hexoside (Suppl. Table 1). Compounds 11, 65 and 75 showed parent peaks at *m/z* 537, 681 and 651, respectively and were recorded as limocitrol-hexoside [M+HCOO]^−^, limocitrol-hexoside HMG and limocitrin-hexoside HMG isomer, respectively. Similarly, compounds 3 and 44 presented deprotonated molecular ion peaks at *m/z* 395 and 315 in ESI –ve ion mode, respectively and were defined as isorhamnetin sulfate and its aglycone, isorhamnetin (3.85%, butanol fraction), respectively. Isorhamnetin showed a demethylated aglycone fragment at *m/z* 301 confirming the compound identity.

Many flavonoid glycosides were characterized from *A. halimus* and can be discussed as follows. Compound 12 presented its parent peak at *m/z* 449 and two fragments appeared at *m/z* 317 and 287 due to the loss of the myricetin aglycone and decarboxylation of the aglycone (Suppl. Table 1) and was found in high percentage in the ethyl acetate (11.20%) fraction, methanol extract (10.00%) and the DCM (5.49%) fraction where it was likely defined as myricetin-pentoside. Similarly, compounds 28, 46 and 74 with deprotonated peaks at *m/z* 271 (+ ve), 579 (-ve) and 465 (-ve), respectively were found to be apigenin, syringetin rutinoside and taxifolin hexoside, respectively. Moreover, compounds 49, 54 and 67 presented parent peaks at *m/z* 507, 725 and 620 in ESI negative ion mode, respectively and were assigned to spinacetin-hexoside ether, apigenin-pentosyl-(hydroxyferuloyl)-pentoside and acacetin-[acetyl-hexosyl-xyloside], respectively.

### Terpenoids

#### Diterpenoids

Two diterpene derivatives were observed and presented in Suppl. Table 1. The deprotonated molecular ion peak at [M-H]^−^ *m/z* 597 was defined as abietatriene-tetrol-hexoside (2.54%, butanol fraction and 4.74%, aqueous fraction). In addition to that, another abietatriene derivative was detected at *m/z* 658 (1.71%, only from the aqueous fraction) and was identified as abietatriene-tetrol-*di*-hexoside.

#### Triterpenoids

In contrast to diterpenoids, triterpenoids represented the second most identified class of phytoconstituents with a total of sixteen compounds (Suppl. Table 1, Fig. 1A, B). Oleanane and oleanoic acid derivatives were the most abundant followed by stigmastadiene and stigmastatriene derivatives. Compound 4 showed a parent peak at [M + H]^+^ *m/z* 471 (2.40%, methanol extract only) and was determined as an oleanane type triterpenoid. Moreover, compound 13 was detected at *m/z* 675 (ESI negative ion mode) (2.44%) only from the total extract and was identified as oleanenoic acid, hexuronic acid ether, methyl ester. Another peak was shown at *m/z* 808 in negative mode from the DCM fraction (1.58%) and was defined as the triterpene, methyl ester, [pentosyl-hexoside] derivative of hydroxyl-oleanenedioic acid.

A parent peak was detected at [M-H]^−^ *m/z* 826 (2.27%, only DCM) and was identified as (heptadecanoyl-hexoside) derivative of stigmastadienol. Moreover, another peak was shown at *m/z* 663 for the triterpene, heptadecanoyl derivative of stigmastatrienol (5.05%, extract, 6.70%, DCM, 5.73%, ethyl acetate) while compound 76, which is also a stigmastane triterpene, had its parent peak at *m/z* 786 (-ve mode) (2.38%, extract) and was assigned to digilanatoside B. In addition to that, the tentative identification of compound 23 as *α*/*β*-amyrin was attributed to the presence of a deprotonated peak in positive mode at *m/z* 427 with 1.40% only from the butanol fraction. The parent peaks for compounds 31 and 42 were detected at *m/z* 721 and 485, respectively and were identified as eicosanoyl derivative of ursenol and alisol C, respectively.

### Phenolic acids

Only three peaks were defined as phenolic acids or their derivatives (Suppl. Table 1). Compounds 37 and 45 were caffeic acid derivatives with peaks at *m/z* 533 and 683, respectively which were found to be caffeic acid hexoside derivative and caffeic acid-hexoside dimer, respectively. Meanwhile, compound 22 was identified as coumaric acid hexoside and it presented its parent peak at *m/z* 325 with characteristic fragment at *m/z* 164 due to loss of coumaroyl moiety. It is worthy noted that, the three phenolic acids were only identified in negative ion mode from the total extract of *A. halimus* (Suppl. Table 1).

### Steroids

A parent peak was detected at [M+HCOO]^−^ *m/z* 525 for the steroid derivative named hydroxyecdysone and it presented daughter fragments at *m/z* 443 and 425 due to loss of water molecules (5.29%, extract, 4.25%, ethyl acetate and 1.25%, DCM). Two other steroids were detected at *m/z* 634 and 381 (both in ESI –ve mode) for pregnenolone 3-sulfate (extract only) and fragment of sterol ester (4.41%, extract and 2.41%, aqueous fraction)^5^, respectively.

### Tannins

#### Hydrolysable tannins

Five peaks were likely identified as hydrolysable tannins (Suppl. Table 1). A deprotonated peak was seen at *m/z* 469 (ESI –ve) with fragments at *m/z* 425, 381 and 275 due to the successive loss of CO_2_ moiety, loss of C_2_O_2_ moiety and ellagic acid-CO (4.71%, ethyl acetate and 2.12%, extract) which was identified as valoneic acid dilactone. Four other peaks were detected and all were classified as galloylated derivatives for compounds 20, 36, 52 and 68 with parent peaks at *m/z* 633, 649, 683 (685) and 619, respectively which were assigned to HHDP-galloyl hexoside (only from the extract), methyl trigalloyl hexose (2.46%, butanol, 2.34%, extract and 2.13%, aqueous), *di*-galloyl hexose (2.58%, extract only) and trigalloyllevohexosan (extract only), respectively.

#### Condensed tannins

A fragment of procyanidin trimer presented its peak at *m/z* 713 (4.04% DCM). Moreover, another condensed tannin named catechin gallate was detected at *m/z* 441 (+ ve) (2.48% from the aqueous extract only) (Suppl. Table 1).

### Phenyl propanoids

Two quinic acid derivatives were detected as compounds 16 and 39. Compound 16 had a parent peak at *m/z* 677 (1.61% extract, 2.24% DCM and 4.17% ethyl acetate fraction) while compound 39 showed its peak at *m/z* 707 and it was detected from the total extract (0.90%), the aqueous fraction (2.46%) and the butanol fraction (3.23%). Compounds 16 and 39 were identified as *di*-caffeoylquinic acid hexoside and *di*-caffeoylquinic acid, respectively. Moreover, a peak was detected at *m/z* 353 in ESI positive ion mode and was defined as the famous phenyl propanoid, chlorogenic acid (7.88% from the butanol fraction only) (Suppl. Table 1).

### Multivariate data analysis using double dendrogram (clustered heat map)

**Fig. 2:**
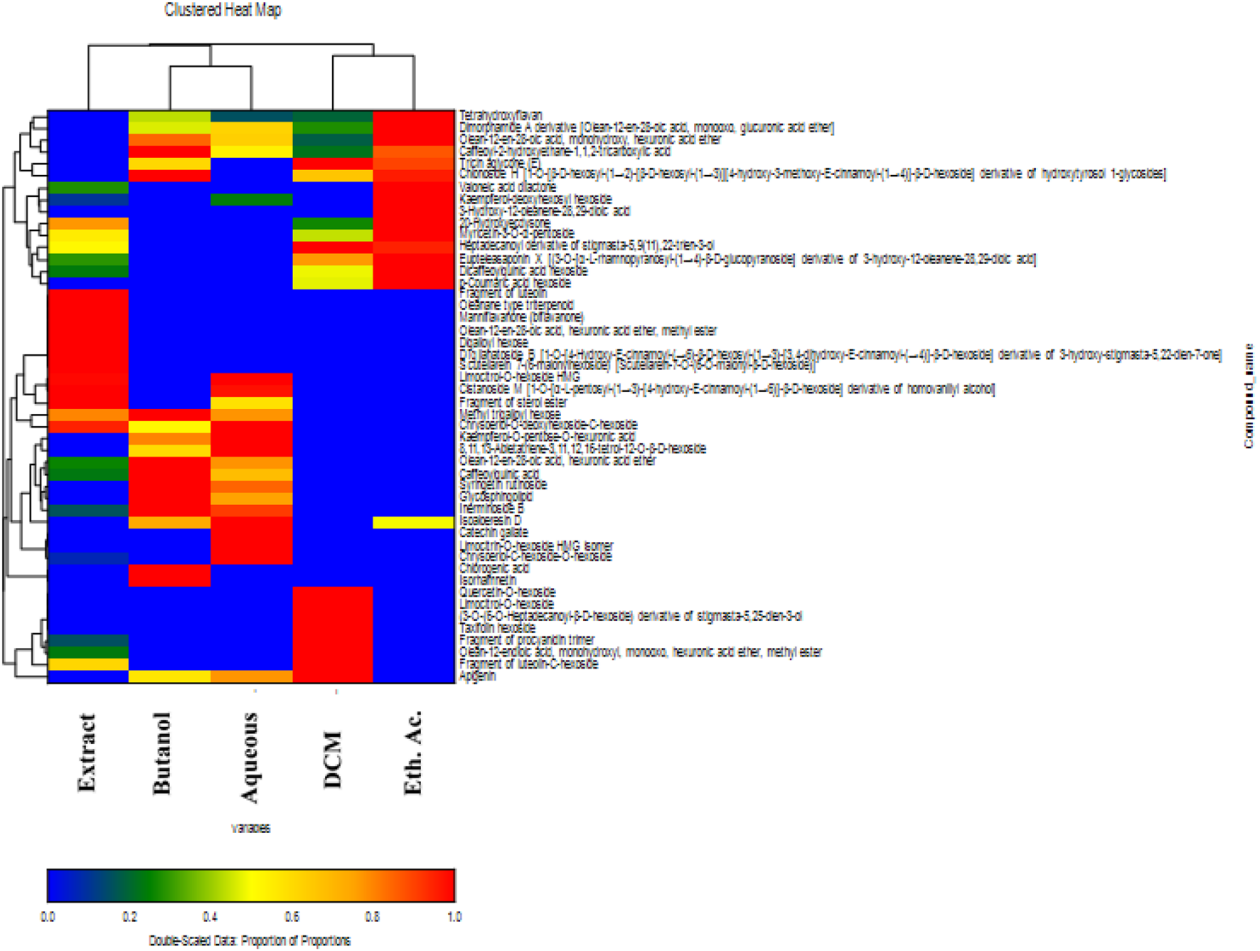
Clustered heat map showing the main tentatively identified compounds from *A. halimus* extract and fractions (compounds with composition ≥ 2% were displayed). [The clustered heat map was constructed using Euclidean distance and the unweighted group method]

Clustered heat map (double dendrogram) was utilized here in this study to show the differences in the phytoconstituents distribution between the total extract of *A. halimus* compoared to the fractions of different polarities. Metabolites with composition ≥ 2% were included in the clustered heat map, the color pattern ranged from blue for the lowest area percentage and the color intensity gets warmer gradually until red for the highest area percentage. As shown in Fig. 2, components with the highest percentage (denoted with red in the clustered heat map) in both the butanol and aqueous fractions were similar in composition thus grouping together while the DCM and ethyl acetate fractions presented similar components with different composition and formed other cluster. Moreover, when comparing the total extract to the fractions, certain similarities were found together with other unique components only found in the total extract (as clarified before in the results section) which lead to its positioning in single cluster (Fig. 2).

### Antimicrobial activity

Data summarized in Suppl. Tables 2 and illustrated in Suppl. Figure S3 indicated that methanol extract had an inhibitory effect only on *Staphylococcus aureus* and *Escherichia coli*, but the latter suffered the strongest effect, since the inhibition zone recorded for them was 12.0 and 30.0 mm, respectively. Correspondingly, the ethyl acetate fraction exhibited a proximate inhibition to that of methanol extract, as it caused 14.0 and 22.0 mm inhibition zone for *S. aureus* and *E. coli*, respectively. On the other hand, butanol had no effect against any of the tested microorganisms, while dichloromethane and water inhibited the growth of only *S. aureus*, where they caused almost a similar inhibition (18.0 and 17.0 mm, respectively).

### Repellent activity

**Fig. 3:**
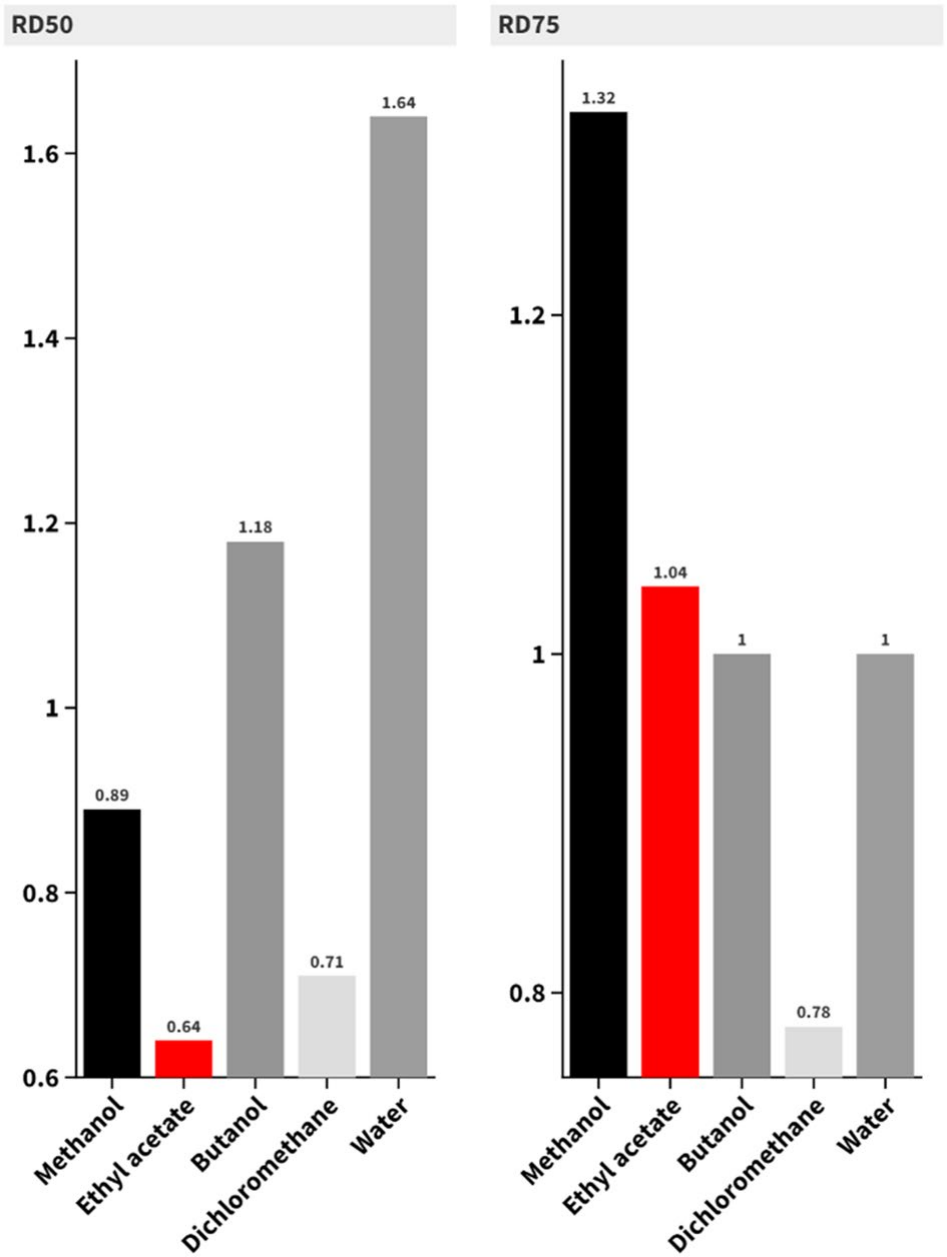
Bar chart showing the repellent activity of *Atriplex halimus* methanol extract and fractions against *Anopheles pharoensis* starved females.

The results from the study examining the repellent activity of *Atriplex halimus* extract and fractions against *Anopheles pharoensis* starved females reveal a potential efficacy among the different extracts. Dichloromethane at 6.67 mg/cm² demonstrated the highest repellency (81.92%) among the tested samples, performing significantly better than other extracts and closely approaching the repellency of the positive control (DEET), which achieved 100% repellency. This indicates that dichloromethane is highly effective as a natural repellent. Butanol fraction at 6.67 mg/cm² also showed strong repellent activity with a repellency rate of 74.49%, making it the second-best natural extract. Ethyl acetate at the same concentration had a lower, but still substantial, repellency of 66.48%. The methanol extract, while effective, showed the lowest repellency among the high-concentration samples (57.76% at 6.67 mg/cm²) (Suppl. Table S3, Fig. 3).

The dose-response pattern was consistent across all samples, where higher concentrations resulted in significantly increased repellency. For example, the repellency of dichloromethane dropped to 36.22% at 1.67 mg/cm², and butanol to 26.83% at the same concentration. The repellency rates increased significantly as concentrations rose, highlighting the concentration-dependent nature of these extracts.

Statistical analysis using ANOVA confirmed significant differences between the treatments (*p* < 0.001), suggesting that the tested samples were not equally effective. Tukey pairwise comparisons further highlighted that dichloromethane and butanol at higher concentrations shared statistical similarities with DEET in their repellency, while the lower concentrations and less effective extracts, like methanol, showed significantly lower performance.

### Molecular modeling

**Fig. 4:**
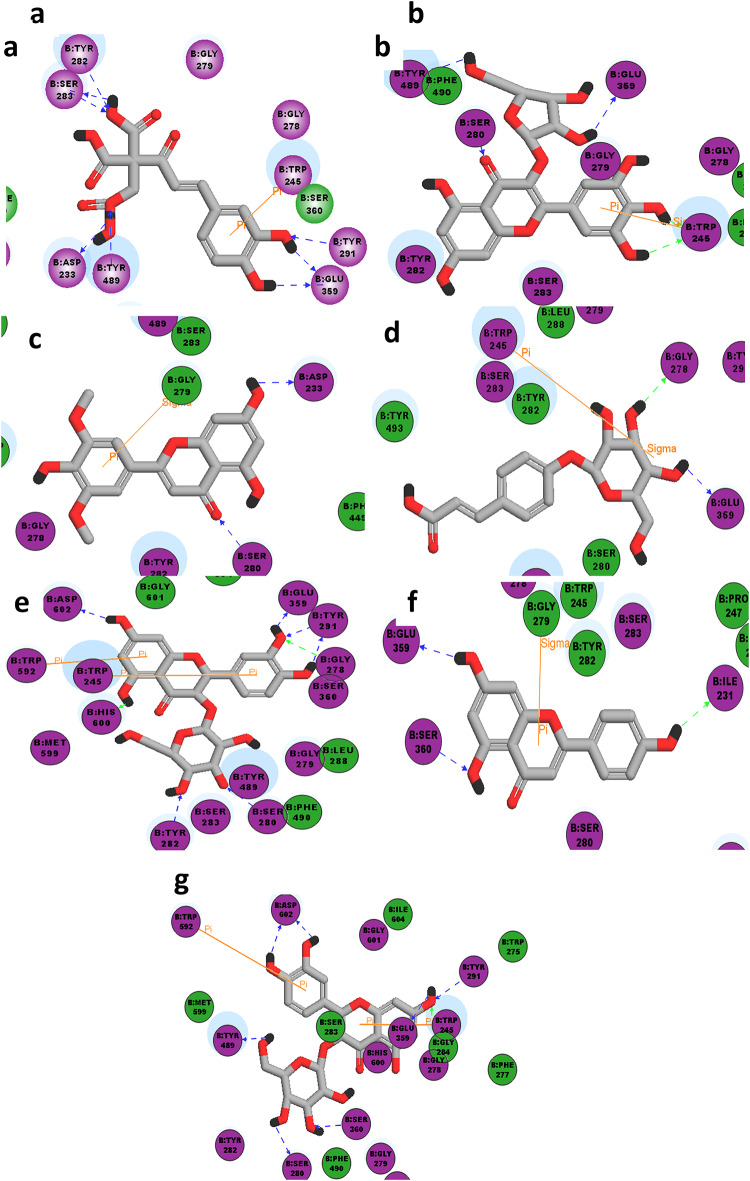
The 2D-interaction diagrams of high score docked compounds with acetylcolinestrase (pdb code: 6ARY) (**a**) _Caffeoyl-2-hydroxyethane-1,1,2-tricarboxylic_acid, (**b**) Myricetin-3-*O-*pentoside, (**c**) Tricin aglycone (E), (**d**) *p*-Coumaric acid hexoside, (**e**) Taxifolin hexoside, (**f**) Apigenin, (**g**) Quercetin-*O*-hexoside.

**Fig. 5:**
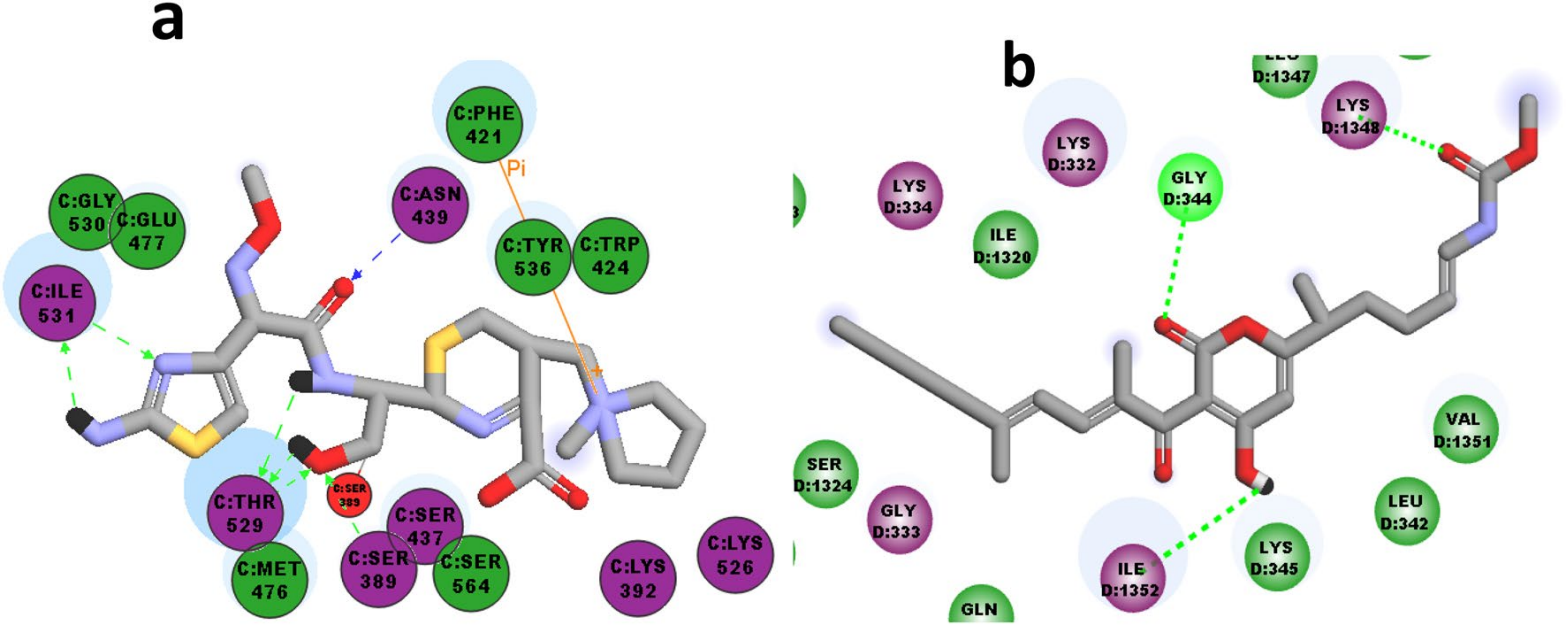
The 2D-interaction diagrams of co-crystallized ligands (**a**) *Staph. aureus*, pdb code: 8CF3 & (**b**) *E.coli*, pdb code: 4YFX.

To get deeper insight into the observed insecticide activity, 9 bioactive compounds present in high proportions in different extracts of *A. halimus* were selected and subjected to molecular docking studies. Interestingly, the binding interactions of the docked compounds at the active site of acetylcholine esterase enzyme (pdb code: 6ARY) were investigated and compared to that of original co-crystallized difluoromethyl ketone inhibitor as previously shown in (Suppl. Table 4, Figs. 4 and 5). Moreover, CDOCKER interaction energies of the docked compounds were recorded and compared to that of the docked co-crystallized ligand (difluoromethyl ketone).

**Fig. 6:**
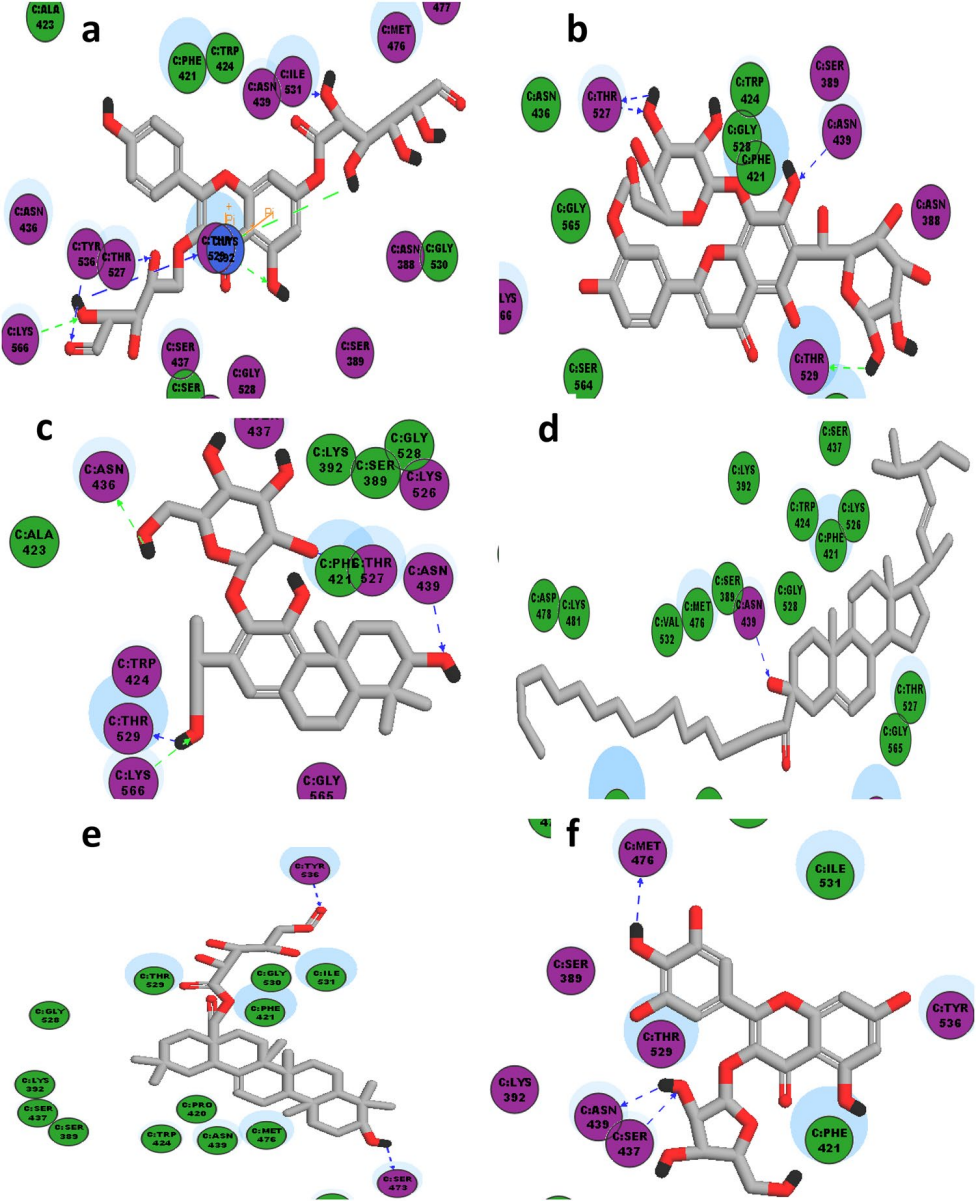

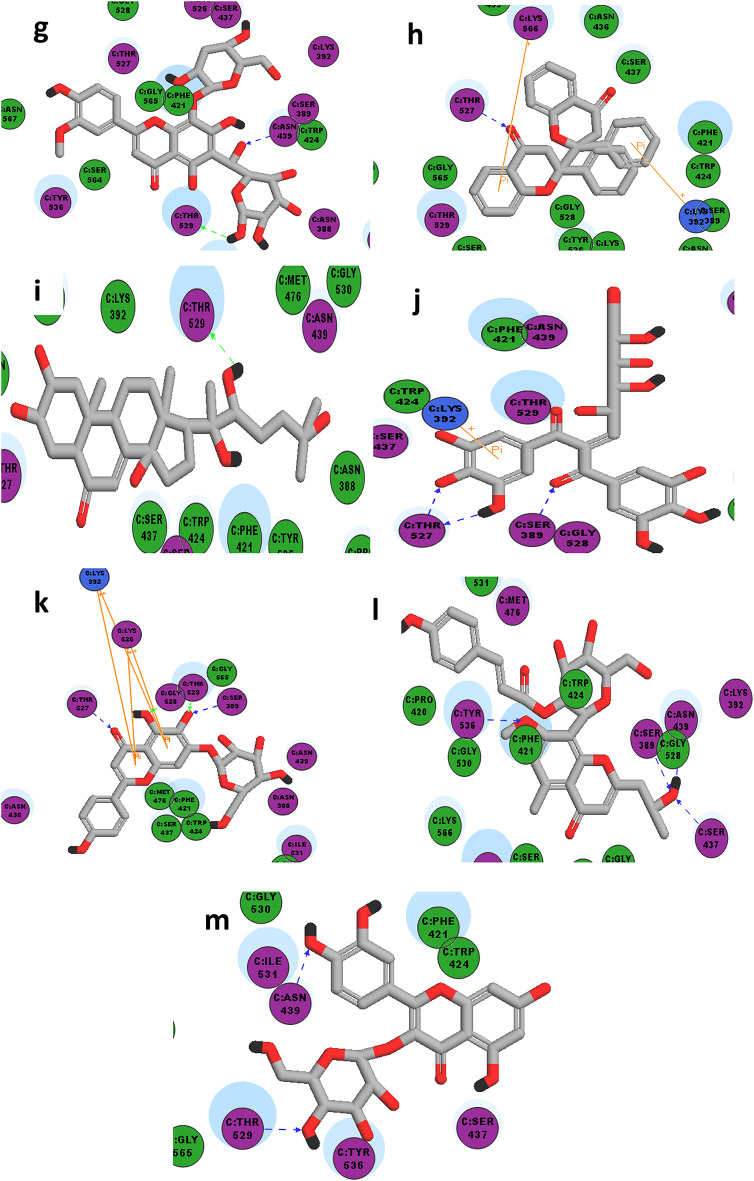
The 2D-interaction diagrams of high score docked compounds with **BLAR sensor domain** (pdb code: **8CF3**) (**a**) Kaempferol-*O*-pentose-*O*-hexuronic_acid, (**b**) Chrysoeriol-*C*-hexoside-*O*-hexoside, (**c**) 8,11,13-Abietatriene-3,11,12,16-tetrol-12-O-β-D-hexoside, (**d**) Heptadecanoyl stigmasta-5_9(11) 22-trien-3-ol), (**e**) Olean-12-en-28-oic acid, monohydroxy, hexuronic acid ether, (**f**) Myricetin-3-*O*-α-pentoside,, (**g**) Chrysoeriol-*O*-deoxyhexoside-*C*-hexoside, (**h**) Manniflavanone (biflavanone), (**i**) 20-Hydroxyecdysone, (**j**) Digalloyl hexose, (**k**) Scutellarein 7-(6-malonylhexoside), (**l**) Isoaloeresin D, (**m**) Quercetin-*O*-hexoside.

**Fig. 7:**
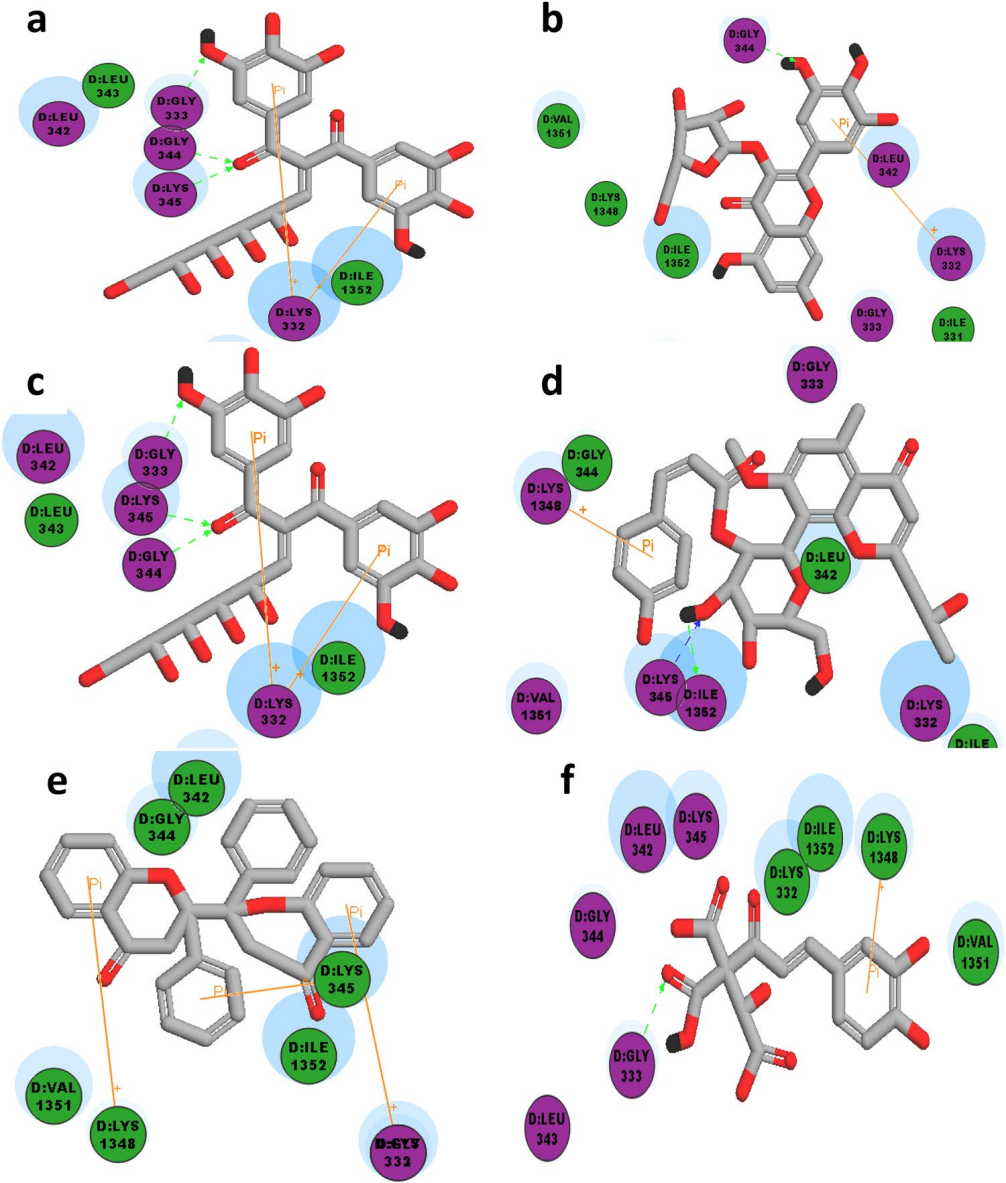
2D-interaction diagrams of high score docked compounds with RNApolymerase (pdb code: **4YFX**) (**a**) _ Digalloyl hexose, (**b**) Myricetin-3-*O*-*α*-pentoside, (**c**) Scutellarein 7-(6-malonylhexoside) [Scutellarein-7-*O*-(6-*O*-malonyl-*β*-D-hexoside)], (**d**) Isoaloeresin_D, (**e**) Manniflavanone (biflavanone), (**f**) Caffeoyl-2-hydroxyethane-1,1,2-tricarboxylic acid.

On the other hand, aiming to rationalize the noted antimicrobial activity in *Staph. aureus* and *E.coli*, 16 bioactive compounds highly available in different *A. halimus* extracts were subjected to molecular docking using the corresponding targets (*Staph. aureus*, pdb code: **8CF3** *and E.coli*, pdb code: **4YFX***)* where the observed CDOCKER interaction energies, as well as the binding interactions of the docked compounds, were compared to those of the original co-crystallized ligand in each corresponding target (Suppl. Table 5, Figs. 6 and 7).

## Discussion

The methanol extract and fractions of varying polarities from *Atriplex halimus* are studied herein to evaluate and compare their phytochemical profiles and to investigate their insecticidal activities. Seventy-eight compounds are tentatively identified where flavonoids and terpenoids constituted the main classes of identified phytoconstituents together with other less abundant components. The extract show unique phytochemical profile compared to the fractions while the two highly polar fractions (the butanol and the aqueous fractions) are quite similar. Moreover, the DCM and the ethyl acetate fractions are matching in some of their components and different in others. Flavonoids being the most abundant class are mainly detected from the methanol extract and the aqueous fraction which can be attributed to the nature of these compounds which favors their presence in the methanol extract and its polar fractions.

On the other hand, *di*-and triterpenoids are traced from the methanol extract and the fractions in near proportions due to the presence of many of them in the form of sugar glycosides which allows their appearance in polar fractions as well as the non-polar ones. Upon reviewing the literature on genus *Atriplex*, many studies are focusing on the LC/MS identification of its phytoconstituents. *A. halimus* was reported to have antioxidant^6^, antiacetylcholinesterase^7^ and hypoglycaemic^8^ activities. The genus is also rich in saponins, alkaloids, betains, proteins, amino acids, mineral salts and phytoecdysteroids^9–11^. Other studies isolate certain components such as quercetin and kaempferol^12^, isorhamnetin, 6-methoxyisorhamnetin and 6-methoxyquercetin^1^.

One study report different components identified from the aerial parts extract of *A. halimus* from Sardinia. The identified compounds were 30,50-dimethoxymyricetin-pentosyl-*O*-pentosyl-hexoside, 30-methoxyquercetin-*O*-pentosyl-hexosyl-*O*-pentosyl-pentoside, 30- methoxyquercetin-*O*-pentosyl-3-*O*-pentosyl-hexoside, 30,50-dimethoxymyricetin-*O*-pentosyl-hexoside, myricetin, quercetin, isorhamnetin glycosides, simple phenolic acids and esters^1^.

In another study, the phytochemical composition of *A. halimus* is investigated through LC/MS where quercetin, and kaempferol were identified. The extract was evaluated against cancer cells using the MTT assay where it had cytotoxic activity at 100 and 200 µg/ml^13^. Different parts of *A. sagittata* were evaluated by LC/ESI/MS^n^. Among the identified phenolic compounds 4-hydroxybenzoic and salicylic acids, kaempferol-3-hexoside-7-pentoside, kaempferol-3-rutinoside are identified^3^.

The metabolic profile, antibacterial and molluscicidal effects of *Atriplex halimus* were evaluated where the extract showed molluscicidal activity against *Biomphalaria alexandrina* (LC_50_=223.8 mg/L), the survival rate of snails exposed to sub-lethal concentrations (LC_25_) reach 20% and snail eggs hatchability significantly reduced^14^. The ethanol extract of *A. halimus* leaves is analyzed through LC/MS^n^ where flavonoids and phenolic acids are among the major components. The extract show significant inhibitory activity in all tested tumor cell lines and the inhibition activity is dose-dependent^15^.

Extracts of *A. laciniata* were tested against human parasites and various pests. The extracts used are crude saponins and solvent samples including methanol, ethyl acetate, choloroform, *n*-hexane and water. Anthelmintic potentials of the samples were analyzed against *Pheretima posthuma* and *Ascaridia galli* using contact toxicity method. Insecticidal activities were performed against *Heterotermes indicola*, *Monomorium pharaonis*, *Tribolium castaneum* and *Rhyzopertha dominica* using standard protocols^16^.

The insecticidal activity of the leaves aqueous and hydroalcohol [methanol/water, 20/80 (v/v)] extracts of *A. halimus* is investigated against *D. opuntiae*. *A. halimus* aqueous extract show the highest activity with mortality rates of 67.04% (after 4 days) and 85% (after 8 days) on nymphs and adult females of *D. opuntiae*, respectively. It also shows the highest mortality rate of nymphs with 100% (4 days after application) and 83.75% of adult females (7 days after the second application) at a concentration of 5% when combined with black soap at 10 g/L under greenhouse conditions. Thirty-six triterpene glycosides together with phytoecdysones and glycosylated phenolic acids and flavonoids were detected from the LC/MS^n^ profiling of *A. halimus*^17^. In addition to that, the acetone/ ethanol, petroleum-ether, ether and chloroform extracts of *A. halimus* are potent against *Culex* larvae (LC_50_ = 115, 36, 54 and 48 ppm, respectively)^18^.

*Atriplex halimus* has been previously reported as a promising source of bioactive secondary metabolites with insecticidal and larvicidal potential, particularly against mosquito vectors such as *Anopheles* spp. The activity of *A. halimus* extracts has been primarily attributed to their abundance of phenolic acids, flavonoids, and related compounds, which are known to disrupt important physiological processes in insects, such as detoxification mechanisms, enzyme inhibition, and larval development disruption. A plausible connection between the chemical profile of *A. halimus* and its possible insecticidal effects is supported by the present study’s findings regarding the dominance of similar metabolite classes among the major and abundant constituents. This correlation highlights the significance of these metabolites for upcoming bioassay-guided validation and vector control studies and offers a mechanistic framework for ranking them as potential contributors to activity against *Anopheles* species^19^.

Our data emphasized that *S. aureus* shows susceptibility to all samples under investigation, except for the the butanol fraction which exhibited no antibacterial effect, whereas the water fraction showed the maximum antibacterial activity. Both the methanol extract and the ethyl acetate fraction exhibited a severe inhibitory impact on *E. coli* compared to their effect on *S. aureus*. These results match those mentioned in earlier studies on ethanolic extracts of *Punica granatum*, *Syzygium aromaticum*, *Zingiber officinales* and *Thymus vulgaris*^20^, and crude ethanol leaf extract of *Calpurnia aurea*^21^. In contrast, all the tested extracts present no antimicrobial activity on *B. spizizenii*, *P. aeruginosa*, and *C. albicans*.

A correlation between the extract’s repellent activity, solvent used in extraction, and the dose of extract used was recorded. The repellent activity of tested extract and fractions reflected the complexity of its chemical components, which may jointly or independently produce the repellent activity^22^. It is worthy noted that the observed repellency activity is predicted or tentative.

Generally, all concentrations from tested extract and fractions evoke repellent activity against *Anopheles pharoensis* starved females. The repellent action of tested extracts can be attributed to the presence of several compounds such as terpenoids, phenolics and alkaloids^23^. The repellent activity exhibited by tested extracts confirms the previously results of *Pyrus communis n-*hexane (PCH) and methanol (PCM) extracts, where at 5.0 mg/cm^2^, PCH recorded the potent repellent activity (95.5%) against *An. pharoensis* females, while PCM recorded 80.0% at the same dose^11,24^ Also, *Galaxaura rugosa* petroleum ether and methanol extracts recorded 85.26 and 77.85% repellent activity against *An. pharoensis* females at 0.67 mg/cm^2^, respectively^10^.

In the current study, molecular modelling technique is used used to support better understanding about the practically observed insect repellant capabilities as well as antimicrobial activities.

Remarkably, all insects roughly exhibit 2 acetylcholinesterases (AChEs) (namely, AChE1 and AChE2)^25^. Moreover, it has been reported that AchE1 enzyme particularly exhibits high structural consistency among different *Anopheles sp*.^26^. Functionally, AchE1 possess a conserved catalytic triad (Ser-His-Glu) located at the active site, which is responsible for the breakdown of acetylcholine into acetate and choline^27^. Interestingly, G119SAchE1 is a mutated form of AchE1 enzyme, where Gly119 residue was replaced with Serine, keeping the catalytic triad conserved unchanged. Yet, this mutation affects the binding of the insecticide to the active site of the enzyme^28^.

Although the exact 3D crystal structure of *An. pharoensis* is not known yet, however, it has been reported that *An. pharoensis* and *An.gambiae* share the same genetic composition^29^. In this context, the published crystal structure of mutated acetylcholine esterase enzyme (G119S AchE1) in *An.gambiae* (**pdb code: 6ARY**) has been used in our molecular modeling studies alternatively to rationalize the insecticide observed practically.

It is worth mentioning that all the chosen nine bioactive compounds subjected to docking against acetylcholine esterase enzyme (**pdb code: 6ARY**) were able to bind and fit into the enzyme active site, which confirms the insecticide activity of all tested extracts. All docked compounds showed higher –CDOCKER interaction energies than original difluoromethyl ketone ligand, except for digalloyl hexose, present in methanol extract only. Regarding the binding site, it was observed that difluromethyl ketone ligand exhibited 3 hydrogen bondings with **Gly 279**, **Ser 280** and **Ala 361**. Furthermore, all the docked compounds showed at least one hydrogen bond with one of the forementioned aminoacids in addition to another unreported interaction, except for caffeoyl-2-hydroxyethane-1,1,2-tricarboxylic_acid and *p*-coumaric acid hexoside which displayed no reported interactions but alternative unreported ones. Interestingly, the superior insecticide activity observed in DCM extract was mostly attributed to the presence of both quercetin-*O*-hexoside and taxifolin hexoside, which are exclusively present in DCM extract. This superior repellency of the DCM extract could be probably attributed to the chemical nature of its constituents. The presence of compounds such as quercetin-*O*-hexoside and taxifolin hexoside, bearing a hydrophobic aglycone which enhanced their lipophilicity compared to the predominant components in the other fractions. Accordingly, these compounds could exhibit enhanced hydrophobic interaction with the insects’ odorant-binding proteins (OBPs), which typically contain hydrophobic binding pockets adapted for sensing semi-polar to non-polar molecules. Furthermore, the moderate volatility of these flavonoid aglycones and associated triterpenoids in the DCM fraction could enhance their diffusion in the environment, allowing them to reach and activate OBPs more effectively. Conversely, highly polar, water-soluble compounds available in the aqueous fraction may be less able to penetrate the hydrophobic OBP cavities, thus reducing their overall repellent activity. Therefore, both the lipophilicity and partial volatility of compounds in the DCM fraction likely contribute to their enhanced bioactivity as insect repellents^30^.

Besides, the decrease in insecticide activity observed using butanol extract compared to that observed in DCM extract could be explained due to the fitting and interaction of 3 compounds only out of the 9 docked compounds, together with the absence of quercetin-*O*-hexoside and taxifolin-hexoside, present in butanol extract. Also, the decrease in insecticide activity recorded using ethyl acetate extract is suggested to be as a result of missing apigenin, present in both DCM and butanol extracts. Unfortunately, 3-hydroxy-12-oleanene-28,29-dioic acid, observed in ethyl acetate extract only, failed to fit into the active site of the enzyme, probably due to its bulkiness. Finally, the least observed activity using methanol extract is supposed to be due to binding of 2 compounds only, among which was digalloyl hexose whose –CDOCKER interaction energies was much less (-21.68) compared to that of the original ligand (-33.36).

With respect to the antimicrobial activity, the extracellular BLAR sensor domain present in *Staphylococcus aureus* (pdb code: **8CF3**) is particularly responsible for resistance to B-lactam antibiotics. Accordingly, the bacterial resistance could be blocked *via* binding of an inhibitor at this sensor domain resulting in restoring the effectiveness of the applied antibiotics^31^.

On the other hand, the gene expression in *E. coli*, as well as, bacterial growth and viability is controlled by RNA polymerase (pdb code: **4YFX**). Therefore, RNA elongation could be inhibited or terminated through the binding of the suitable inhibitor at RNA polymerase binding site, which would subsequently affect bacterial growth and survival^32^.

The 2D interaction diagram of BlaR sensor domain in *Staph. aureus* (pdb code: **8CF3)** bound to its original ligand (cefepime) showed hydrogen bonding with Ser 389, Asn 439, Thr 529 and Ile 531, in addition to pi-pi interaction with Phe 421. Whereas the 2D interaction diagram RNA polymerase in *E.coli* (pdb code: **4YFX)** bound to its original ligand (Myxopyronin B) showed essential hydrogen bonding with Gly344 in addition to hydrogen bonding with other aminioacids as Lys 1348 and Ile 1352.

The potent antimicrobial activity against *Staph. aureus* observed using DCM extract may be attributed to the exclusive presence of high proportion of quercetin-*O*-hexoside which showed 2 hydrogen bonding with 2 reported aminoacid residues (Asn 439 and Thr 529) in addition to a comparable –CDOCKER interaction energy to that of the co-crystallized lead compound as mentioned in Table 5.

The significant antimicrobial activity against *Staph. aureus* observed using water extract is likely due to the presence of chrysoeriol*-C-*hexoside*-O-*hexoside in water extract only in very high proportion. Remarkably, this compound showed higher –CDOCKER interaction energy than the original ligand in addition to its hydrogen bonding with the essential Asn 439.

Noteworthy, the antimicrobial activity of chrysoeriol-*O*-deoxyhexoside-*C*-hexoside, present in both butanol and water extracts, is observed in water extract according to its abundancy, as it is observed in double proportion in water extract compared to its proportion in butanol extract.

Moreover, the antimicrobial activities against *Staph. aureus* using either methanol or ethylacetate extracts are comparable to that of the reference tetracycline could by best explained due to the presence of at least three highly available compounds in each extract with higher –CDOCKER interaction energies compared to the ligand and capable of hydrogen bonding with at least one reported or unreported aminoacid residue.

Also, the the exclusive availability of manniflavanone exclusively present in the methanol extract is supposed to contribute to its antimicrobial activity antimicrobial activity although it shows a different mode of interaction and binding with unreported aminoacid residues.

Interestingly, the molecular docking results of the current study suggest that the superior antimicrobial activity of methanol extract against *E.coli* is probably due to the presence of digalloyl hexose and manniflavanone exclusively in methanol extract. Moreover, these two compounds are able to fit well at the active site of RNA polymerase and bind with at least one of the reported aminoacids. Additionally, digalloyl hexose shows higher –CDOCKER interaction energy (-46.68) and manniflavanone shows almost similar –CDOCKER interaction energy (-38.43) compared to the original ligand (-40.90).

Furthermore, the relatively similar antimicrobial activity observed in *E.coli* using ethyl acetate extract compared to the standard tetracycline could be suggested due to the presence of Myricetin-3-O-α-pentoside in high proportion similar to that in methanol extract. Notably, the aforementioned compound exhibits higher –CDOCKER score compared to that of the original lead compound and successfully interacted *via* hydrogen bonding with the essential Gly 344 in addition to another unreported pi-pi interaction with Lys 332.

Finally, the absence of antimicrobial activity observed using DCM, butanol and water extract could be explained as result of several factors such as: the reduced availability of compounds showing higher –CDOCKER interaction energies compared to the original ligand such as myricetin-3-*O*-α-pentoside in DCM extract, or the much reduced –CDOCKER interaction energies of some docked compounds remarkably abundant especially in butanol and water extracts such apigenin and Olean-12-en-28-oic acid, monohydroxy, hexuronic acid, in addition to the failure of these docked compounds with reduced –CDOCKER interaction energies to interact with any of the essential aminoacids at the active site.

## Methods

### Plant collection

Fresh aerial parts of *Atriplex halimus* were collected from El-Orman botanical garden, Giza, Egypt [Latitude: **~** 30.0292° N, Longitude: **~** 31.2131° E] (during Autumn 2022). The plant sample was identified and botanically authenticated by agricultural engineer Terease Labib, Consultant of Plant Taxonomy at the Ministry of Agriculture, El-Orman Botanical Garden, and National Gene Bank, Giza, Egypt. All plant materials used in this study were collected and handled in full compliance with relevant institutional, national, and international guidelines and legislation. The plant species investigated are not classified as endangered or at risk of extinction, and therefore do not fall under restrictions outlined by international conservation frameworks. Furthermore, the study adheres to the principles of the *IUCN Policy Statement on Research Involving Species at Risk of Extinction* and the *Convention on the Trade in Endangered Species of Wild Fauna and Flora (CITES)*. No protected or regulated species were collected, and all necessary considerations regarding biodiversity conservation and ethical research practices were strictly followed. Dried voucher specimen was deposited at the Herbarium of Pharmacognosy Department, Faculty of Pharmacy, Ain Shams University, Cairo, Egypt under the code: PHG-P-AH-585.

### Chemicals

Analytical grade solvents *viz*. methanol, dichloromethane (DCM), ethyl acetate and butanol were utilized in the extraction and fractionation and were purchased from Sigma-Aldrich^®^, Cairo, Egypt.

### Plant extract preparation and fractionation

The dried aerial parts of *Atriplex halimus* (1 kg) were comminuted to a suitable size and extracted by maceration in 70% methanol using a plant-to-solvent ratio of 1:5 (w/v). The mixture was macerated at room temperature for 48 h with occasional stirring, and then filtered. This extraction cycle was repeated three times until exhaustion of the plant material. The combined filtrates were concentrated under reduced pressure and evaporated to dryness. The dried extract (42 g) was then lyophilized and stored in a moisture-free container. The total extract was defatted with petroleum ether and subsequently subjected to successive solvent fractionation using dichloromethane (20 g), ethyl acetate (3 g), *n*-butanol (3 g), and water (8 g). Each fraction was evaporated under reduced pressure to remove the solvent, lyophilized, and stored for further analysis.

### Ultra performance liquid chromatography–electrospray ionization- mass spectrometry (UPLC/ESI/MS) analysis

UPLC/ESI/MS in both positive and negative ion acquisition modes was carried out on a XEVO TQD triple quadrupole instrument, Waters Corporation, Milford, MA 01757, U.S.A., mass spectrometer^4^. Chromatographic separation of the sample was done by injecting 10 µl into a UPLC instrument equipped with a reverse-phase C-18 column (ACQUITY UPLC—BEH, 2.1 × 50 mm column; 1.7 *μ*m particle size). The sample (100 *µ*g/mL) solution was prepared using HPLC-grade methanol, filtered using a membrane disc filter (0.2 *μ*m), degassed by sonication before injection, and then subjected to LC/ESI/MS analysis. The gradient mobile phase comprises two eluents: eluent A is H₂O acidified with 0.1% formic acid, and eluent B is MeOH acidified with 0.1% formic acid. Elution was made at a flow rate of 0.2 mL/min as follows: (10% B) from 0 to 5 min.; (30% B) from 5 to 15 min.; (70% B) from 15 to 22 min.; (90% B) from 22 to 25 min.; and (100% B) from 25 to 29 min. The analysis was accomplished using negative ion mode as follows: source temperature 150 °C, cone voltage 30 eV, capillary voltage 3 kV, desolvation temperature 440 °C, cone gas flow 50 L/h, and desolvation gas flow 900 L/h. Mass spectra were recorded in electrospray ionization (ESI) (negative and positive ion modes) (m/z 100–1000). UPLC/MS data were processed using Masslynx 4.1 software, and tentative identification was done by comparing their retention times (Rt), mass spectra, and fragmentation patterns with reported data. Level 1 (confirmed identification): Compounds identified by comparison with authentic standards, based on matching accurate mass, retention time, and MS/MS fragmentation patterns. Level 2 (putatively annotated compounds): Compounds annotated using accurate mass and MS/MS spectral matching against public metabolite databases (e.g., METLIN, MassBank, HMDB) in the absence of reference standards. Level 3 (putatively characterized compound classes): compounds assigned to chemical classes based on diagnostic fragments and characteristic spectral features. Level 4 (unknown compounds): reproducible features detected without sufficient information for structural annotation.

### Multivariate data analysis using clustered heat map

For the construction of the clustered heat map, NCSS. 12 software with Euclidean distance and the unweighted pair group method was used. Both samples and metabolites were clustered based on similarity in abundance patterns, allowing the identification of common, unique, and differentially accumulated metabolites across samples.

Hierarchical clustering analysis (HCA) visualized as clustered heat map was performed on the normalized dataset and visualized as a clustered heat map to assess similarities and differences in metabolite abundance profiles among samples. Clustering was conducted using an appropriate distance metric (e.g., Euclidean distance) and linkage method, enabling the identification of common and distinct metabolic patterns across the analyzed samples.

### Antimicrobial activity assay

Disc diffusion method^33^ was used to test samples antimicrobial activity in vitro against a wide range of pathogens, including Gram positive bacteria; *Bacillus spizizenii* ATCC 6633, *Staphylococcus aureus* ATCC 6538, Gram negative bacteria; *Pseudomonas aeruginosa* ATCC 9027, *Escherichia coli* ATCC 8739, and unicellular fungi; *Candida albicans* ATCC 10,231. Sterile paper discs (6 mm diameter) were impregnated with (20 µL) of the tested samples at a concentration of (10 mg/mL), allowed to dry under aseptic conditions, and then placed onto the surface of inoculated agar plates. In accordance with CLSI guidelines^34^, inhibition zones were determined after 24 h of incubation for bacteria and 3 days for fungi at 37 °C on the plates. The antimicrobial activity was evaluated by measuring the diameter of the inhibition zones (including the disc diameter) in millimeters using a calibrated ruler. All experiments were performed in triplicate, and the results were expressed as mean inhibition zone diameters ± standard deviation.

### Repellent activity assay

#### Tested *Anopheles pharoensis*

*Anopheles pharoensis* larvae were collected from Faiyum Governorate (Latitude: 29º18 × 53.4’’ N, Longitude: 30º39 × 19.2’’ E, Elevation: 19 m), Egypt. The collected larvae were reared for several generations under controlled conditions of temperature (25–27 °C), relative humidity (60–80%) and photoperiod (12 L:12D). A standard rearing procedure followed to provide larvae needed for the bioassay^35^. This study did not involve vertebrate animals; experiments were performed on *Anopheles pharoensis* (Diptera: Culicidae), and therefore ethical approval was not required.

#### Repellent activity test

The repellent activity was carried out following a procedure described with small modifications^10^. Three doses of each tested extract or fraction (6.67, 3.33, and 1.67 mg/cm^2^) were prepared in 2 ml of solvent with two drops of Tween_80_. Each solvent with 2 drops of Tween_80_ were used as controls. Positive control (DEET) was purchased from a commercial pharmacy. Standard cages (60 × 60 × 80 cm) used to test the repellent activity against *A. pharoensis* starved females. The dose was directly applied onto 5 × 6 cm of ventral surface of pigeon after feathers removal from the abdomen. After 10 min, the treated pigeons were placed in the cages containing fifty *A. pharoensis* starved females (5- 7d-old) for three hours. Three replicates were usually used for each dose along with the control. Three replicates were usually used along with the control. The repellency percentages were calculated using a standard formula^36^. All experimental protocols involving pigeons were reviewed and approved by the Department of Zoology, Medical Entemology, Faculty of Science, Al-Azhar University (boys). While the study was categorized as a low-risk, non-invasive observational trial. The procedures were carried out under the supervision of qualified researchers in accordance with local veterinary standards and institutional norms for animal care. The work was conducted in the spirit of the Helsinki Declaration and the ARRIVE guidelines for reporting animal research.

#### Statistical analysis

Data were calculated as Mean ± SD. ANOVA was used to evaluate the data, as recommended^37^. The SPSS V.22 was used for data encoding and entry. Quantitative data were reported using mean, and standard deviation; qualitative data were presented with frequency. The threshold for statistical significance was set at *P* < 0.05. Analysis evaluated using MiniTab V 14. Data were visualized when possible, using R Studio V 2022.02.4.

### Molecular modeling studies

The molecular docking study in the current study was performed using Accelrys’s Discovery Studio 2.5.5 software (Accelrys Inc., San Diego, CA, USA) at the Faculty of Pharmacy, Ain Shams University, Egypt. The 3D structures of the different targets used in our docking studies were obtained from the Protein Data Bank at the Research Collaboration for Structural Bioinformatics (**RCSB**) website (www.rcsb.org). (acetylcholine esterase in *Anopheles sp* pdb code: **4ARY**, *E. coli* pdb code: **4YFX** and *S. aureus* pdb code: **8CF3).**

The 3D structures of the forementioned targets were then uploaded to Accelrys’s Discovery Studio 2.5.5 software, followed by adding hydrogen atoms and energy calculation by applying force field followed by energy minimization. Before minimization, fixed atom constrains were created to avoid the distortion of the original 3D structure during minimization.

Simultaneously, the target compounds (the highly available compounds in different extracts) were prepared before docking using (Prepare ligands) protocol where the energies of the compounds were minimized.

Afterwards, the prepared target compounds together with the minimized target were subjected to the docking study using CDOCKER protocol. After the docking run is completed, the CDOCKER interaction energies were recorded and the binding interactions of the docked ligands with the critical aminoacids at the active site were investigated and compared to those of the original ligand in each target.

## Conclusion

The methanol extract and fractions of *Atriplex halimus* aerial parts were evaluated through UPLC/MS analysis where seventy-eight components were detected. The annotated components were from the flavonoids and triterpenoids as the main phytochemical classes. *A. halimus* 70% methanol extract and fractions that were evaluated in the present study represented new repellent agents against the malaria vector, *Anopheles pharoensis.* Noteworthy, the observed insecticide activity in addition to antimicrobial activities were well rationalized using the molecular docking studies which aided to get better idea about the binding of the bioactive compounds into the binding site of the corresponding targets. Finally, more studies are needed to evaluate the insecticidal activity of *A. halimus* against other several mosquito species and to pinpoint the main components responsible for such activity.

## Supplementary Information

Below is the link to the electronic supplementary material.

Supplementary Material 1

Supplementary Material 2

## Data Availability

All the data supporting this article have been included in the research article. If the raw data is required, it will be made available on request from the first author.
