# Supplementary material for: Phytochemical profiling, metabolomics, and molecular docking studies of Atriplex halimus aerial parts revealing potential insecticidal activity against the malaria vector Anopheles pharoensis
Source: Sci Rep. 2026 May 21;16:15880. doi: 10.1038/s41598-026-52695-1 (PMC13194954; doi:10.1038/s41598-026-52695-1)
Supplement: Supplementary file 1 — Supplementary Material 1 [file 41598_2026_52695_MOESM1_ESM.docx]

**Supplementary file**

**Phytochemical profiling, metabolomics, and molecular docking studies of Atriplex halimus aerial parts revealing potential insecticidal activity against the malaria vector Anopheles pharoensis**

**Esraa A. Elhawary^1*^, Hassan O. Waheeb^2^, Abeer H.A. Abdelhafiz^3,^ Amr A. El-Waseif^4^, Ahmed Z.I. Shehata^5*^**

^1^Department of Pharmacognosy, Faculty of Pharmacy, Ain Shams University, Cairo 11566, Egypt.

^2^Department of Zoology, Faculty of Science (Boys), Al-Azhar University, Cairo 11651, Egypt.

^3^Department of Pharmaceutical Chemistry, Faculty of pharmacy, Ain Shams University, Cairo 11566, Egypt

^4^Botany and Microbiology Dept., Faculty of Science (Boys), Al-Azhar University, Cairo 11651, Egypt.

**Corresponding authors:**

***Esraa A. Elhawary, Assistant Professor of Pharmacognosy,** Department of Pharmacognosy, Faculty of Pharmacy, Ain Shams University, Cairo 11566, Egypt. [Esraa.elhawary@pharma.asu.edu.eg](mailto:Esraa.elhawary@pharma.asu.edu.eg). Tel: 002-01005195311

***Ahmed Z.I. Shehata,** Associate Professor of Medical Entemology, Department of Zoology, Faculty of Science (Boys), Al-Azhar University, Cairo, Egypt. [ahmed.ibrahem84@azhar.edu.eg](mailto:ahmed.ibrahem84@azhar.edu.eg) Tel: 002-01028802244

| **Suppl. Table. 1. UPLC/MS analysis results showing the tentatively identified metabolites from *Artiplex halimus* 70% methanol extract and fractions**  **No.** | **Compound name** | **Molecular Formula** | **Class** | **R_t_ (min.)** | **[M-H]^-^**  **(*m/z*)** | **[M+H]^+^**  **(*m/z*)** | **Source**  **(% Composition)** | | | | | **Ref.** |
| --- | --- | --- | --- | --- | --- | --- | --- | --- | --- | --- | --- | --- |
|  |  |  |  |  |  |  | **Ext.** | **DCM** | **Eth.**  **Ac.** | **Bu.** | **Water** |  |
| 1 | Tetrahydroxyflavan | C_15_H_14_O_5_ | Flavonoid | 0.68 | **273** | **-** | 0.74 | 1.48 | **4.91** | **3.25** | 1.75 | ^1^ |
| 2 | Fragment of luteolin | - | Flavonoid | 1.22 | **-** | **311** | 18.08 | - | - | - | - | ^2^ |
| 3 | Isorhamnetin sulfate | C_16_H_12_O_10_S | Flavonoid | 1.46 | **395** | **-** | 0.71 | - | - | - | - | ^3^ |
| 4 | Oleanane type triterpenoid | - | Triterpene | 1.85 | **-** | **471** | 2.40 | - | - | - | - | ^4^ |
| 5 | Manniflavanone (biflavanone) | C_30_H_22_O_13_ | Flavonoid | 2.02 | **-** | **591** | 2.63 | - | - | - | - | ^5^ |
| 6 | Valoneic acid dilactone | C_21_H_10_O_13_ | Hyd. Tannin | 2.73 | **469** | **-** | 2.12 | - | **4.71** | - | - | ^6^ |
| 7 | Unknown Fragment | - | - | 3.09 | **425** | **-** | 0.87 | 1.29 | - | - | - | - |
| 8 | Quercetin-hexoside | C_21_H_20_O_12_ | Flavonoid | 6.01 | **463** | **-** | - | **12.46** | - | - | - | ^7^ |
| 9 | Hydroxyecdysone | C_27_H_44_O_7_ | Steroid hormone | 6.07 | **525** | **-** | **5.29** | 1.25 | **4.25** | - | - | ^3^ |
| 10 | Salvianolic acid A | C_26_H_22_O_10_ | Stilbenoid polypropanoid | 6.31 | **-** | **495** | - | - | 0.51 | - | - | ^8^ |
| 11 | Limocitrol-hexoside | C_24_H_26_O_14_ | Flavonoid | 6.61 | **537** | **-** | - | 2.17 | - | - | - | ^9^ |
| 12 | Myricetin-pentoside | C_20_H_18_O_12_ | Flavonoid | 6.80 | **449** | **-** | **10.00** | **5.49** | **11.20** | - | - | ^3^ |
| 13 | Oleanenoic acid, hexuronic acid ether, methyl ester | C_37_H_60_O_9_ | Triterpene | 7.83 | **675** | **-** | 2.44 | - | - | - | - | ^3^ |
| 14 | (Heptadecanoyl-hexoside) derivative of stigmastadienol | C_52_H_90_O_7_ | Triterpene | 7.93 | **826** | **-** | - | 2.27 | - | - | - | ^10^ |
| 15 | Heptadecanoyl derivative of stigmastatrienol | C_46_H_78_O_2_ | Triterpene | 8.07 | **663** | **-** | **5.05** | **6.70** | **5.73** | - | - | ^11^ |
| 16 | *di*-caffeoyl-quinic acid hexoside | C_31_H_36_O_18_ | Phenyl propanoid | 8.19 | **677** | **-** | 1.61 | 2.24 | **4.17** | - | - | ^12^ |
| 17 | Fragment of procyanidin trimer | - | Cond. Tannin | 8.35 | **713** | **-** | 0.91 | **4.04** | - | - | - | ^7^ |
| 18 | Methyl ester, [pentosyl-hexoside] derivative of hydroxy-oleanenedioic acid | C_43_H_68_O_14_ | Triterpene | 8.37 | **808** | **-** | - | 1.58 | - | - | - | ^13^ |
| 19 | Unranoside C | C_39_H_52_O_21_ | Miscellaneous | 8.42 | **856** | **-** | - | 1.66 | - | - | - | ^14^ |
| 20 | HHDP-galloyl hexoside | C_42_H_30_O | Hyd. Tannin | 8.52 | **633** | **-** | 0.72 | - | - | - | - | ^3,15^ |
| 21 | Tricin aglycone (E) | C_17_H_14_O | Flavonoid | 9.31 | **329** | **-** | - | **5.65** | **4.56** | **4.19** | - | ^16^ |
| 22 | Coumaric acid hexoside | C_15_H_18_O_8_ | Phenolic acid | 9.53 | **325** | **-** | - | 2.68 | **5.08** | - | - | ^16^ |
| 23 | *α*/*β*-Amyrin | C_30_H_50_O | Triterpene | 9.57 | **-** | **427** | - | - | - | 1.40 | - | ^1^ |
| 24 | Fragment of luteolin-hexoside | - | Flavonoid | 9.64 | **-** | **455** | 1.81 | 2.03 | - | - | - | ^8^ |
| 25 | Oleanendioic acid, monohydroxyl, monooxo,hexuronic acid ether, methyl ester | C_37_H_60_O_9_ | Triterpene | 9.74 | **691** | **-** | 0.95 | 2.76 | - | - | - | ^3^ |
| 26 | Kaempferol-pentose-hexuronic acid | C_27_H_30_O_15_ | Flavonoid | 9.76 | **617** | **-** | - | - | - | 2.43 | **3.39** | ^16^ |
| 27 | Eupteleasaponin X [pentosyl-hexoside] derivative of hydroxy-oleanenedioic acid] | C_42_H_66_O_14_ | Triterpene | 9.89 | **794** | **817** | 2.17 | **3.95** | **4.62** | - | - | ^13^ |
| 28 | Apigenin | C_15_H_10_O_5_ | Flavonoid | 10.18 | **-** | **271** | - | 7.66 | - | 5.32 | 8.06 | ^8^ |
| 29 | Kaempferol-deoxyhexosyl hexoside | C_27_H_30_O_14_ | Flavonoid | 10.23 | **593** | **645** | 0.88 | - | **5.41** | - | 1.98 | ^1^ |
| 30 | Coumaroyl-caffeoyl-methylpentanoic acid-hydroxy-quinate | - | Miscellaneous | 10.40 | **693** | **-** | 0.68 | - | - | - | - | ^16^ |
| 31 | Eicosanoyl derivative of Ursenol | C_50_H_88_O_2_ | Triterpene | 10.78 | **721** | **-** | 0.57 | - | - | - | - | ^17^ |
| 32 | Oleanenoic acid, hexuronic acid ether | C_38_H_60_O_10_ | Triterpene | 10.89 | **631** | **-** | 1.71 | - | - | **5.56** | **4.82** | ^3^ |
| 33 | Dimorphamide A derivative [Oleanenoic acid, monooxo, hexuronic acid ether] | C_36_H_36_N_2_O_8_ | Triterpene | 10.93 | **645** | **-** | **3.94** | **3.39** | **4.62** | **4.68** | **5.74** | ^3^ |
| 34 | Chlorogenic acid | C_16_H_18_O_9_ | Phenyl propanoid | 11.46 | **-** | **353** | - | - | - | **7.88** | - | ^16,18^ |
| 35 | Oleanenoic acid, monohydroxy, hexuronic acid ether | C_36_H_58_O_9_ | Triterpene | 11.68 | **647** | **649** | 2.11 | 2.30 | **5.46** | **6.54** | **5.96** | ^3^ |
| 36 | Methyl *tri*-galloyl hexose | C_27_H_24_O_18_ | Hyd. Tannin | 12.64 | **649** | **-** | 2.34 | - | - | 2.46 | 2.13 | ^19^ |
| 37 | Caffeic acid hexoside derivative | - | Phenolic acid | 12.86 | **533** | **-** | 0.84 | - | - | - | - | ^20^ |
| 38 | Acetylated kaempferol deoxyhexosyl hexoside | C_29_H_34_O_17_ | Flavonoid | 12.94 | **-** | **635** | 0.64 | - | - | - | - | ^1^ |
| 39 | Caffeoylquinic acid | C_16_H_18_O_9_ | Phenyl propanoid | 13.09 | **707** | **-** | 0.90 | - | - | **3.23** | 2.46 | ^21^ |
| 40 | Kaempferol-*di*-deoxyhexoside  (Kaempferitrin) | C_27_H_30_O_14_ | Flavonoid | 13.73 | **577** | **-** | 0.98 | - | - | - | 1.74 | ^1^ |
| 41 | Quercetin pentosyl hexoside | C_26_H_28_O_16_ | Flavonoid | 13.85 | **595** | **-** | - | - | - | - | 1.86 | ^1^ |
| 42 | Alisol C | C_30_H_46_O_5_ | Triterpene | 14.13 | **485** | **-** | - | - | - | - | 1.68 | ^16^ |
| 43 | Quercetin-acetyl-hexoside | C_23_H_22_O_13_ | Flavonoid | 14.16 | **505** | **553** | 1.62 | - | - | - | - | ^22^ |
| 44 | Isorhamnetin | C_16_H_12_O_7_ | Flavonoid | 14.20 | **315** | **-** | - | - | - | **3.85** | - | ^3^ |
| 45 | Caffeic acid-hexoside dimer | C_15_H_18_O | Phenolic acid | 14.55 | **683** | **-** | 0.64 | - | - | - | - | ^16^ |
| 46 | Syringetin rutinoside | C_29_H_34_O_17_ | Flavonoid | 14.83 | **579** | **-** | - | - | - | 2.78 | 2.63 | ^1^ |
| 47 | Scutellarein-pentosyl-pentoside | C_26_H_28_O_14_ | Flavonoid | 14.92 | **564** | **-** | 0.71 | - | - | - | 1.94 | ^23^ |
| 48 | Abietatriene-tetrol-hexoside | C_26_H_40_O_9_ | Diterpene | 15.31 | **597** | **-** | - | - | - | 2.54 | **4.74** | ^24^ |
| 49 | Spinacetin, hexoside ether | C_22_H_22_O_13_ | Flavonoid | 15.32 | **507** | **509** | 1.58 | - | - | - | - | ^3^ |
| 50 | Isoaloeresin D | C_29_H_32_O_10_ | Anthraquinone | 15.76 | **555** | **-** | - | - | **4.17** | **8.46** | **12.89** | ^25^ |
| 51 | Feruloyl-harpagide | C_25_H_32_O_13_ | Irridoid glycoside | 15.77 | **540** | **-** | 1.87 | - | - | - | - | ^16^ |
| 52 | *di*-Galloyl hexose | C_20_H_20_O_14_ | Hyd. tannin | 16.27 | **483** | **485** | 2.58 | - | - | - | - | ^16^ |
| 53 | Fragment of luteolin-hexoside-deoxyhexoside | - | Flavonoid | 16.85 | **-** | **473** | - | - | - | - | 0.77 | ^8^ |
| 54 | Apigenin-pentosyl-(hydroxyferuloyl)-pentoside | C_41_H_45_O_18_ | Flavonoid | 17.78 | **725** | **-** | 0.84 | - | - | - | - | ^26^ |
| 55 | Catechin gallate | C_22_H_18_O_10_ | Cond. Tannin | 19.73 | **-** | **441** | - | - | - | - | 2.48 | ^27^ |
| 56 | Caffeoyl-hydroxyethane-tricarboxylic acid | C_14_H_12_O_10_ | Miscellaneous | 20.10 | **339** | **-** | - | 1.47 | **5.11** | **7.96** | **4.79** | ^28^ |
| 57 | Luteolin derivative | - | Flavonoid | 20.19 | **737** | **-** | 0.87 | - | - | - | - | ^29^ |
| 58 | Chrysoeriol-hexoside-hexoside | C_28_H_34_O_17_ | Flavonoid | 20.30 | **-** | **625** | 1.29 | - | - | - | 16.93 | ^8^ |
| 59 | Chrysoeriol-deoxyhexoside-hexoside | C_28_H_32_O_15_ | Flavonoid | 20.88 | **607** | **609** | **5.16** | - | - | 2.38 | **5.09** | ^8^ |
| 60 | Tricin-deoxyhexosyl-hexoside | C_29_H_36_O_16_ | Flavonoid | 21.34 | **-** | **639** | 0.81 | - | - | - | - | ^8^ |
| 61 | Abietatriene-tetrol-*di*-hexoside | C_32_H_50_O_14_ | Diterpene | 24.97 | **658** | **-** | - | - | - | - | 1.71 | ^24^ |
| 62 | Pregnenolone-sulfate | C_21_H_32_O_5_S | Steroid | 25.02 | **634** | **-** | 0.82 | - | - | - | - | ^30^ |
| 63 | Hizivaide B | C_44_H_88_O_6_ | Miscellaneous | 25.15 | **697** | **-** | 0.78 | - | - | - | - | ^31^ |
| 64 | derivative of hydroxytyrosol-glycosides | C_31_H_38_O_17_ | Miscellaneous | 25.29 | **682** | **-** | 0.98 | - | - | - | - | ^32^ |
| 65 | Limocitrol-hexoside HMG | C_24_H_28_O_15_ | Flavonoid | 25.50 | **681** | **-** | 2.06 | - | - | - | 1.95 | ^9^ |
| 66 | Inerminoside B | C_31_H_46_O_16_ | Irridoid glycoside | 25.88 | **674** | **-** | 0.72 | - | - | **3.75** | **3.81** | ^33^ |
| 67 | Acacetin-[acetyl-hexosyl-pentoside] | C_29_H_32_O_15_ | Flavonoid | 26.01 | **620** | **-** | 0.79 | - | - | - | - | ^34^ |
| 68 | *tri*-Galloyl-levo-hexosan | C_27_H_24_O_18_ | Hyd. Tannin | 26.11 | **619** | **-** | 0.94 | - | - | - | - | ^19^ |
| 69 | derivative of homovanillyl alcohol | C_30_H_38_O_14_ | Miscellaneous | 26.91 | **622** | **-** | 2.01 | - | - | - | 1.84 | ^35^ |
| 70 | Fragment of sterol ester | - | Steroid | 28.19 | **381** | **-** | **4.41** | - | - | - | 2.41 | ^36^ |
| 71 | Glycosphingolipid | - | Miscellaneous | 28.35 | **820** | **-** | - | - | - | 2.62 | 2.20 | ^37^ |
| 72 | dimethyl ester derivative of hydroxy-oleanene-dioic acid | C_32_H_50_O_5_ | Triterpene | 28.56 | **514** | **-** | - | 1.65 | - | - | - | ^13^ |
| 73 | Hydroxy-oleanenedioic acid | C_30_H_46_O_5_ | Triterpene | 28.80 | **486** | **-** | - | - | **5.17** | - | - | ^13^ |
| 74 | Taxifolin hexoside | C_21_H_22_O_12_ | Flavonoid | 28.90 | **465** | **-** | - | 2.08 | - | - | - | ^38^ |
| 75 | Limocitrin-hexoside HMG isomer | C_24_H_28_O_15_ | Flavonoid | 29.11 | **651** | **-** | - | - | - | - | **3.17** | ^9^ |
| 76 | Digilanatoside B  [derivative of 3-hydroxy-stigmasta-5,22-dien-7-one] | C_38_H_42_O_18_ | Triterpene | 29.13 | **786** | **-** | 2.38 | - | - | - | - | ^39^ |
| 77 | Scutellarein-(malonyl-hexoside) | C_24_H_22_O_14_ | Flavonoid | 30.47 | **534** | **-** | 2.90 | - | - | - | - | ^23^ |
| 78 | Chionoside H | C_36_H_48_O_21_ | Miscellaneous | 31.07 | **816** | **-** | - | **8.25** | **10.74** | **15.18** | - | ^32^ |
|  | **% Identification** | | | | | | **79.20%** | **76.8%** | **89.91%** | **79.48%** | **77.59** % |  |

**R_t_: retention time, min.: minutes, Ext.: 70% methanol extract, DCM: dichloromethane fraction, Eth. Ac.: ethyl acetate fraction, Bu.: butanol fraction**

**Suppl. Table 2: Inhibition zones presented by *Atriplex halimus* 70% methanol extract and fractions**

| **Test strains** | **Inhibition zone diameter (mm) of samples at concentrations (µg)** | | | | | |
| --- | --- | --- | --- | --- | --- | --- |
|  | **Methanol** | **Eth. Ac.** | **Bu.** | **DCM** | **Water** | **Tetracycline (TE30) Standards** |
| ***Bacillus spizizenii* ATCC6633** | 0.0 | 0.0 | 0.0 | 0.0 | 0.0 | 0.0 (R) |
| ***Staphylococcus aureus* ATCC 6538** | 12 | 14 | 0.0 | 18 | 17 | 14 |
| ***Pseudomonas* *aeruginosa* ATCC 9027** | 0.0 | 0.0 | 0.0 | 0.0 | 0.0 | 0.0 (R) |
| ***Escherichia coli* ATCC 8739** | 30 | 22 | 0.0 | 0.0 | 0.0 | 20 |
| ***Candida albicans* ATCC 10231** | 0.0 | 0.0 | 0.0 | 0.0 | 0.0 | 0.0 (R) |

**Ext.: 70% methanol extract, DCM: dichloromethane fraction, Eth. Ac.: ethyl acetate fraction, Bu.: butanol fraction.**

**Suppl. Table 3:** **Repellent activity of *A. halimus* methanol extract and fractions against *Anopheles pharoensis* starved females**

| **Sample** | **Doses**  **(mg/cm^2^)** | **Unfed females**  **%** | **Repellency %** | **RD_50_**  **Mean**  **(LCL-UCL)** | **RD_75_**  **Mean**  **(LCL-UCL)** | ***χ2*** | |
| --- | --- | --- | --- | --- | --- | --- | --- |
|  |  |  |  |  |  | **RD_50_** | **RD_75_** |
| **Ext.** | 6.67 | 58.0±5.29 | 57.76±4.88 | 5.50  (4.21-6.78) | 8.77  (6.45-11.09) | 0.89^NS^ | 1.32^NS^ |
|  | 3.33 | 38.67±4.16 | 38.27±3.72 |  |  |  |  |
|  | 1.67 | 17.33±1.16 | 16.76±2.14 |  |  |  |  |
| **Eth. Ac.** | 6.67 | 66.67±4.62 | 66.48±4.29 | 4.29  (4.0-4.58) | 7.49  (6.05-8.94) | 0.64^NS^ | 1.04^NS^ |
|  | 3.33 | 49.33±3.06 | 48.97±3.66 |  |  |  |  |
|  | 1.67 | 24.67±3.06 | 24.15±3.34 |  |  |  |  |
| **Bu.** | 6.67 | 74.67±1.16 | 74.49±1.33 | 3.81  (3.29-4.32) | 6.54  (6.42-6.67) | 1.18^NS^ | 1.0^NS^ |
|  | 3.33 | 50.0±4.0 | 49.66±4.04 |  |  |  |  |
|  | 1.67 | 27.33±2.31 | 26.83±2.85 |  |  |  |  |
| **DCM** | 6.67 | 82.0±5.29 | 81.92±5.16 | 2.43  (1.023-3.84) | 5.49  (4.57-6.42) | 0.71^NS^ | 0.78^NS^ |
|  | 3.33 | 66.67±7.02 | 66.44±7.07 |  |  |  |  |
|  | 1.67 | 36.67±3.06 | 36.22±3.68 |  |  |  |  |
| **Water** | 6.67 | 71.33±5.03 | 71.14±5.01 | 4.02  (3.29-4.75) | 6.71  (5.36-8.05) | 1.64^NS^ | 1.0^NS^ |
|  | 3.33 | 54.67±3.06 | 54.38±2.59 |  |  |  |  |
|  | 1.67 | 20.67±1.16 | 20.14±0.24 |  |  |  |  |
| **Positive Control (DEET)** | 1.80 | 0.0 | 100.0±0.0 | --- | --- | --- | --- |
| **Control** | --- | 0.0 | 0.0 | 0.0 | 0.0 | --- | --- |

Number of tested females = 50 per each replicate.

**Suppl. Table 4: Docking results for the docked compounds against acetyl cholinesterase in *Anopheles sp*. (pdb code: 6ARY) and their availability in different extracts**

| **Compound** | **C-DOCKER interaction energy(-ve)** | **Extract**  **(methanol)** | **DCM** | **Ethyl acetate** | **Butanol** |
| --- | --- | --- | --- | --- | --- |
| **Difluoromethyl ketone ligand** | **33.36** |  |  |  |  |
| **Quercetin-*O*-hexoside** | **37.43** |  | **√** |  |  |
| **Tricin aglycone (E)** | **47.98** |  | **√** | **√** | **√** |
| ***p*-Coumaric acid hexoside** | **45.63** |  | **√** | **√** |  |
| **3-Hydroxy-12-oleanene-28,29-dioic acid** | **------** |  |  | **√** |  |
| **Taxifolin hexoside** | **41.15** |  | **√** |  |  |
| **Caffeoyl-2-hydroxyethane-1,1,2-tricarboxylic acid** | **51.10** |  | **√** | **√** | **√** |
| **Myricetin-3-*O*-*α*-pentoside** | **48.30** | **√** | **√** | **√** |  |
| **Apigenin** | **40.76** |  | **√** |  | **√** |
| **Digalloyl hexose** | **21.68** | **√** |  |  |  |

**Suppl. Table 5: Docking results for the docked compounds against the extracellular BLAR sensor domain in *Staph. aureus* and RNA polymerase in *E.coli* and their availability in different extracts.**

| **Compound** | **C-DOCKER interaction energy(-ve)**  ***Staph. aureus*** | **C-DOCKER interaction energy(-ve)**  ***E.coli*** | **Extract**  **(methanol)** | **Ethyl acetate** | **DCM** | **Butanol** | **Water** |
| --- | --- | --- | --- | --- | --- | --- | --- |
| Ligand (**4YFX**) (Myxopyronin B) |  | 40.90 |  |  |  |  |  |
| Ligand (**8CF3**)  (Cefepime) | 48.06 |  |  |  |  |  |  |
| Digalloyl hexose | 41.79 | 46.68 | √ |  |  |  |  |
| Myricetin-3-*O*-*α*-pentoside | 46.77 | 41.88 | √ | √ | √ |  |  |
| Scutellarein 7-(6-malonylhexoside) [Scutellarein-  7-*O*-(6-*O*-malonyl-*β*-D-hexoside)] | 45.71 | 40.25 | √ |  |  |  |  |
| Isoaloeresin_D | 47.53 | 39.69 |  | √ |  | √ | √ |
| Manniflavanone (biflavanone) | 37.45 | 38.43 | √ |  |  |  |  |
| Caffeoyl-2-hydroxyethane-1,1,2-tricarboxylic acid | 27.24 | 38.11 |  | √ | √ | √ | √ |
| Heptadecanoyl stigmasta-5_9(11) 22-trien-3-ol | 46.21 | 32.23 | √ | √ | √ |  |  |
| 20-Hydroxyecdysone | 38.98 | 31.30 | √ | √ | √ |  |  |
| 8,11,13-Abietatriene-3,11,12,16-tetrol-12-*O*-*β*-D-hexoside | 54.35 | 31.12 |  |  |  | √ | √ |
| Chrysoeriol-*O*-deoxyhexoside-*C*-hexoside | 45.23 | 29.64 |  |  |  | √ | √ |
| Olean-12-en-28-oic acid, hexuronic acid ether | 29.96 | 25.75 |  |  |  | √ | √ |
| Dimorphamide A derivative [Olean-12-en-28-oic acid, monooxo, hexuronic acid ether | 27.32 | 24.65 | √ | √ | √ | √ | √ |
| Olean-12-en-28-oic acid, monohydroxy, hexuronic acid ether | 43.66 | 23.87 | √ | √ | √ | √ | √ |
| Kaempferol-*O*-pentose-*O*-hexuronic_acid | 66.67 | 23.06 |  |  |  | √ | √ |
| Chrysoeriol-*C*-hexoside-*O*-hexoside | 52.04 | 22.13 |  |  |  |  | √ |
| Apigenin | 27.64 | 22.07 |  |  | √ | √ | √ |
| Quercetin-*O*-hexoside | 47.02 | 25.91 |  |  |  |  |  |


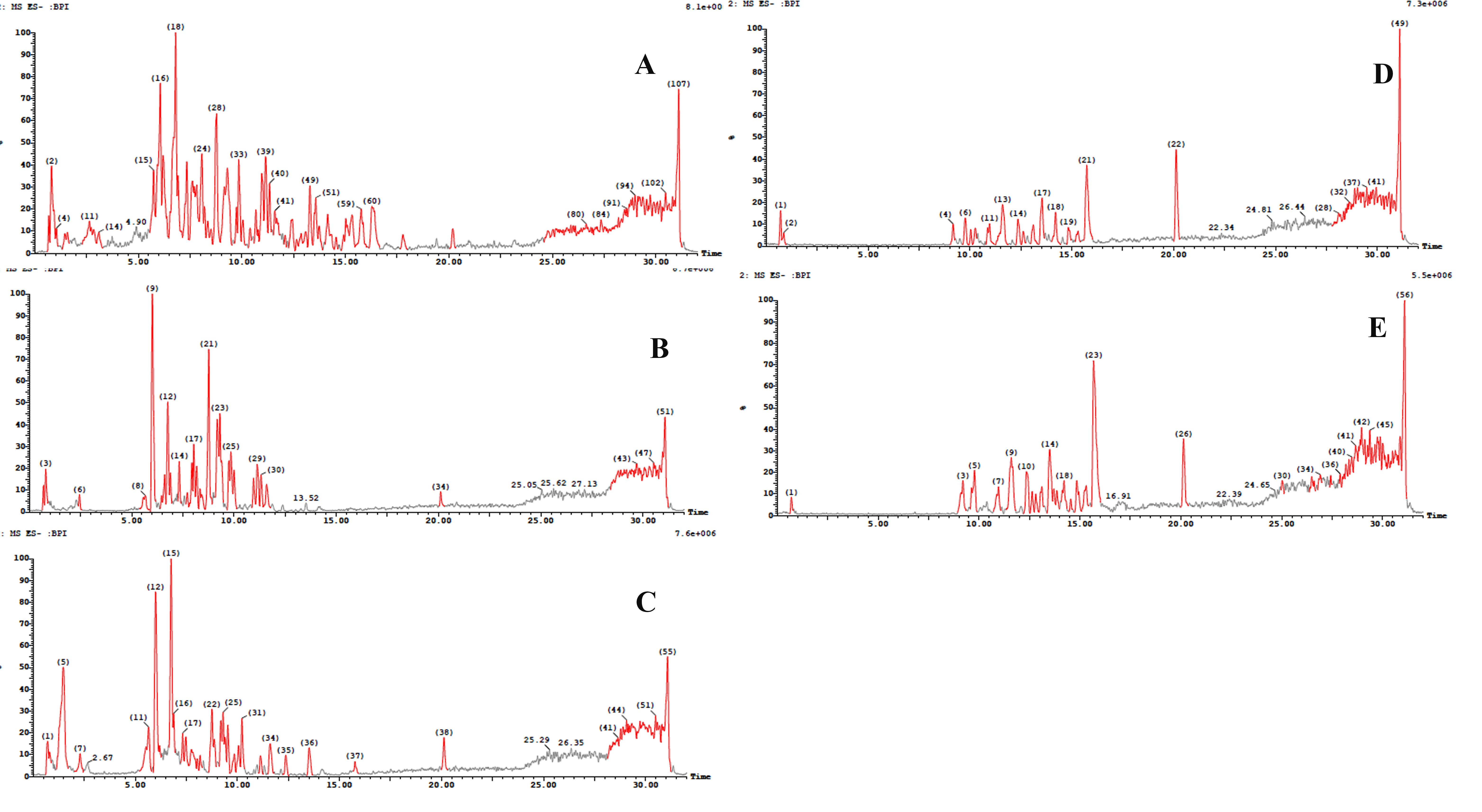


**Suppl. Fig. 1. BPI chromatograms in negative ion mode for *Atriplex halimus* A) methanol extract, B) Dichloromethane fraction (DCM), C) Ethyl acetate fraction, D) Butanol fraction and E) Aqueous fraction.**


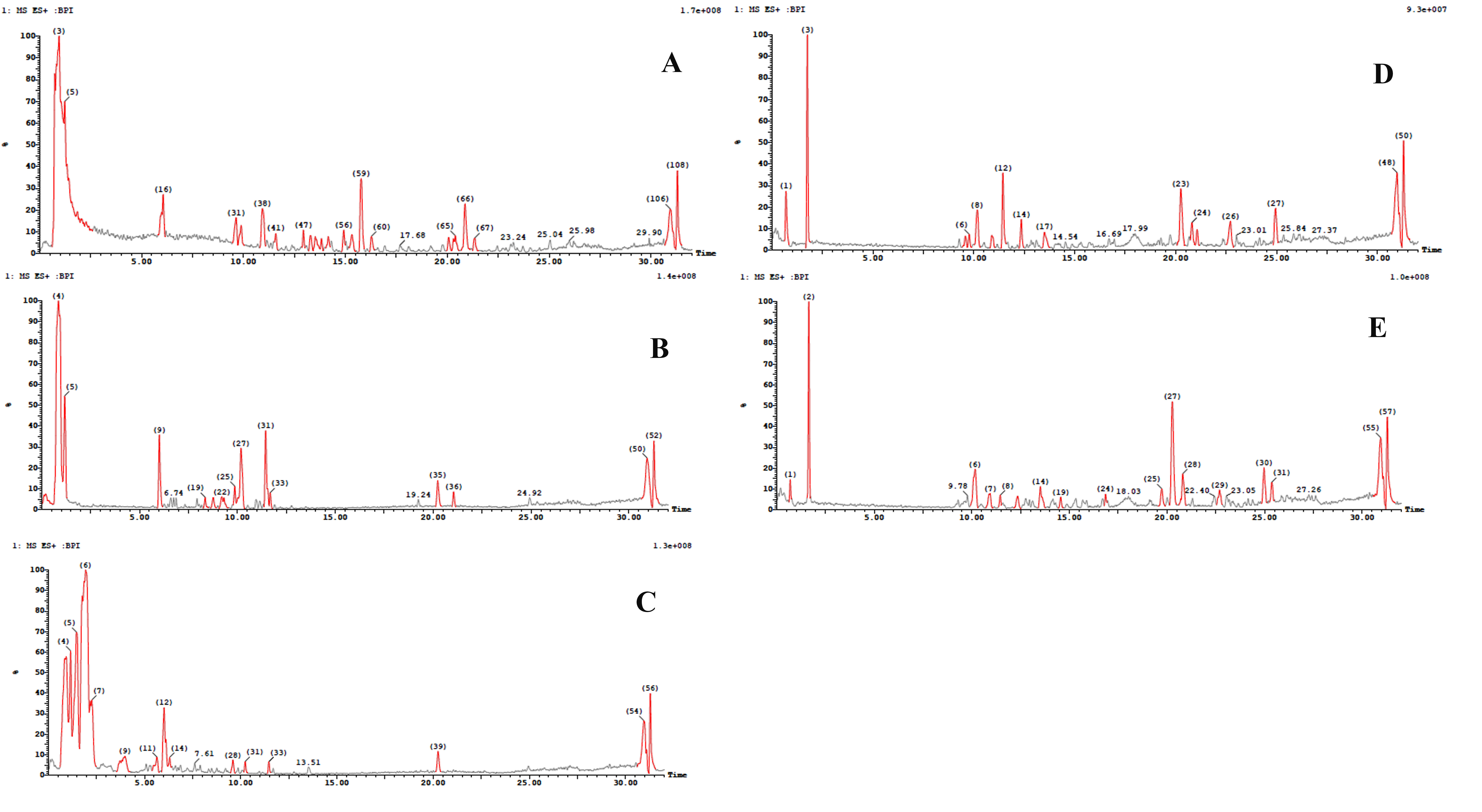


**Suppl. Fig. 2. BPI chromatograms in positive ion mode for *Atriplex halimus* A) methanol extract, B) Dichloromethane fraction (DCM), C) Ethyl acetate fraction, D) Butanol fraction and E) Aqueous fraction.**


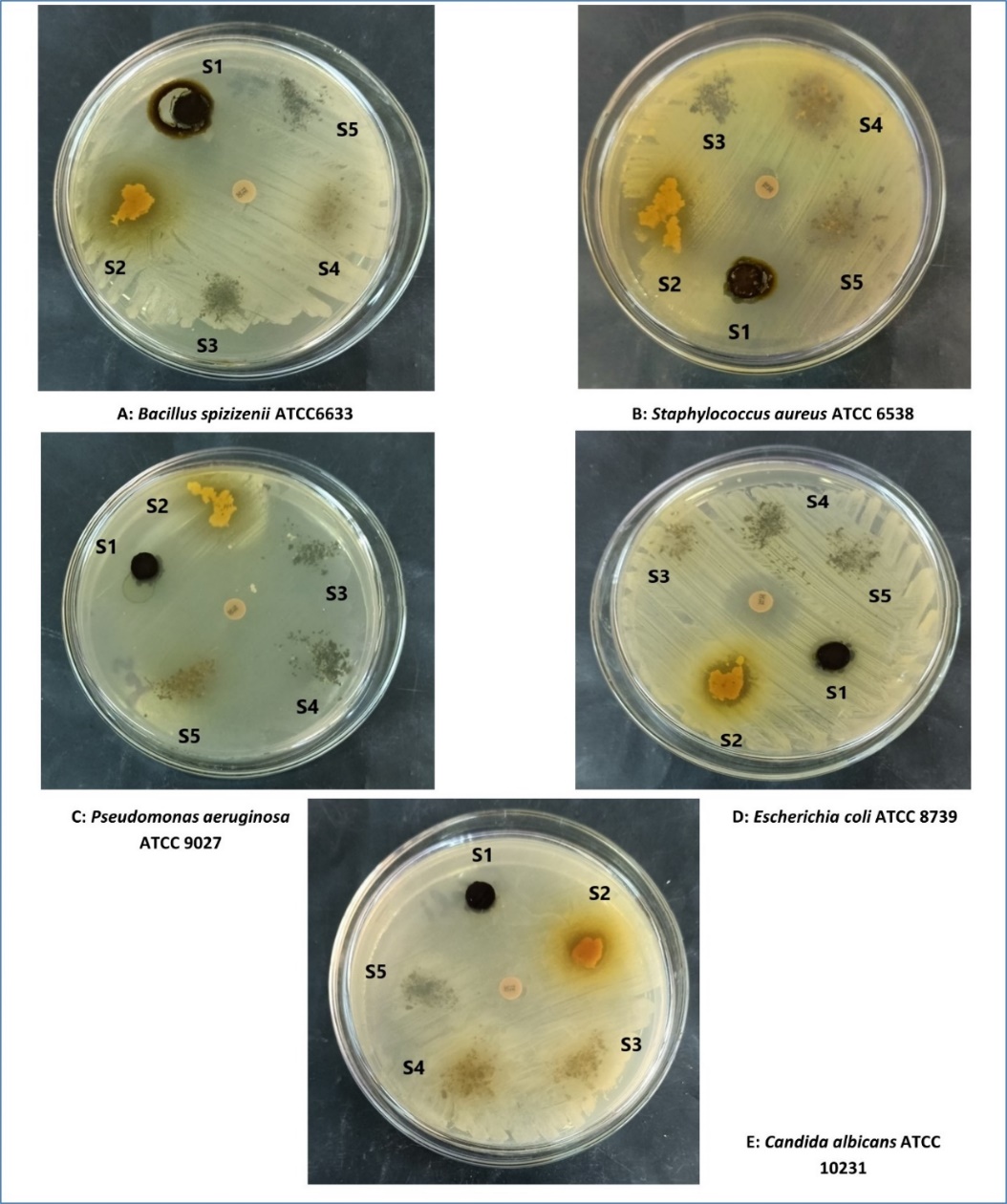


**Suppl. Fig. 3.** The inhibition zone (mm) of the tested extract and fractions against *Bacillus spizizenii* (A), *Staphylococcus aureus* (B), *Pseudomonas aeruginosa* (C), *Escherichia coli* (D) and *Candida albicans* (E). S1: methanol extract, S2: ethyl acetate fraction, S3: butanol fraction, S4: dichloromethane fraction, and S5: water fraction.

**
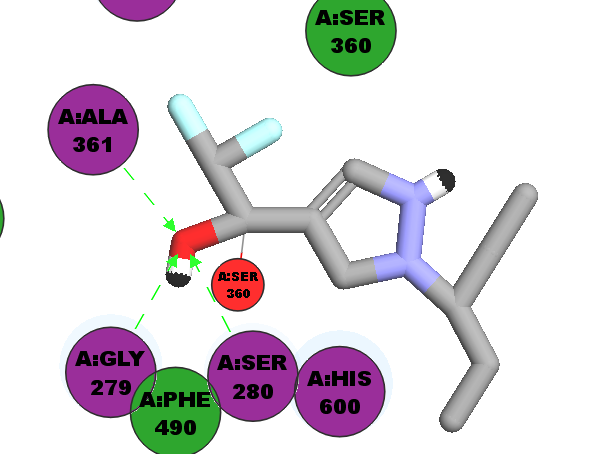

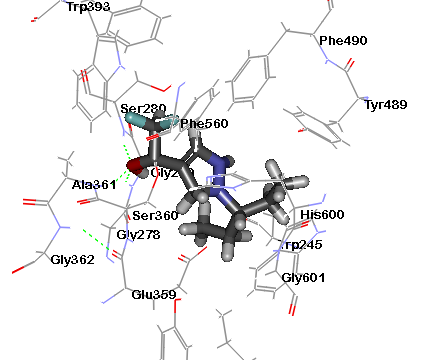
**

**Suppl. Fig. 4. Interaction diagram of ligand co-crystallized with acetylcolinestrase (pdb code: 6ARY) (a) 2D-representaion & (b) 3D-representation**

**References**

1 Zaghloul, E. *et al.* Phytoecdysteroids and Anabolic Effect of Atriplex dimorphostegia: UPLC-PDA-MS/MS Profiling, In Silico and In Vivo Models. *Plants* **12**, 206 (2023).

2 Llorent-Martínez, E. J., Spínola, V., Gouveia, S. & Castilho, P. C. HPLC-ESI-MSn characterization of phenolic compounds, terpenoid saponins, and other minor compounds in Bituminaria bituminosa. *Industrial Crops and Products* **69**, 80-90 (2015).

3 Naboulsi, I. *et al.* Insecticidal Activities of Atriplex halimus L., Salvia rosmarinus Spenn. and Cuminum cyminum L. against Dactylopius opuntiae (Cockerell) under Laboratory and Greenhouse Conditions. *Insects* **13**, 930 (2022).

4 Salih, E. Y. *et al.* LC-MS/MS tandem mass spectrometry for analysis of phenolic compounds and pentacyclic triterpenes in antifungal extracts of Terminalia brownii (Fresen). *Antibiotics* **6**, 37 (2017).

5 Reed, K. A. *Identification of phenolic compounds from peanut skin using HPLC-MSn*, Virginia Tech, (2009).

6 Wyrepkowski, C. C. *et al.* Characterization and quantification of the compounds of the ethanolic extract from Caesalpinia ferrea stem bark and evaluation of their mutagenic activity. *Molecules* **19**, 16039-16057 (2014).

7 Odah, S. M., Salama, M. M., Aziz, W. M., El-Alfy, T. S. & Ezzat, S. M. Anti-wrinkle activity and UPLC-MS/MS Metabolic Profiling of Pomegranate Grape Seeds Extracts. *IJSPR* **11**, 3679-3689 (2020).

8 Wojakowska, A., Perkowski, J., Góral, T. & Stobiecki, M. Structural characterization of flavonoid glycosides from leaves of wheat (Triticum aestivum L.) using LC/MS/MS profiling of the target compounds. *Journal of Mass Spectrometry* **48**, 329-339 (2013).

9 El-Sayed, M. A., Al-Gendy, A. A., Hamdan, D. I. & El-Shazly, A. M. Phytoconstituents, LC-ESI-MS profile, antioxidant and antimicrobial activities of Citrus x limon L. Burm. f. cultivar variegated pink lemon. *Journal of Pharmaceutical Sciences and Research* **9**, 375 (2017).

10 Wang, P. *et al.* A novel C29 sterol from Clerodendrum cyrtophyllum. *Chemistry of Natural Compounds* **48**, 594-596 (2012).

11 Wright, A. D. GC-MS and NMR analysis of Phyllidiella pustulosa and one of its dietary sources, the sponge Phakellia carduus. *Comparative Biochemistry and Physiology Part A: Molecular & Integrative Physiology* **134**, 307-313 (2003).

12 Kramberger, K. *et al.* HPLC-DAD-ESI-QTOF-MS determination of bioactive compounds and antioxidant activity comparison of the hydroalcoholic and water extracts from two Helichrysum italicum species. *Metabolites* **10**, 403 (2020).

13 Youssef, F. S. *et al.* Metabolomics-based profiling of Clerodendrum speciosum (Lamiaceae) leaves using LC/ESI/MS-MS and in vivo evaluation of its antioxidant activity using Caenorhabditis elegans model. *Antioxidants* **11**, 330 (2022).

14 Ragasa, C. Y., Alimboyoguen, A. B. & Shen, C.-C. Chemical constituents of Ficus nota. *Der Pharma Chemica* **6**, 98-101 (2014).

15 Sang, S. & Yang, C. S. Structural identification of novel glucoside and glucuronide metabolites of (-)-epigallocatechin-3-gallate in mouse urine using liquid chromatography/electrospray ionization tandem mass spectrometry. *Rapid communications in mass spectrometry : RCM* **22**, 3693-3699, doi:10.1002/rcm.3786 (2008).

16 Elhawary, E. A., Mostafa, N. M., Shehata, A. Z., Labib, R. M. & Singab, A. N. B. Comparative study of selected Rosa varieties’ metabolites through UPLC-ESI-MS/MS, chemometrics and investigation of their insecticidal activity against Culex pipiens L. *Jordan Journal of Pharmaceutical Sciences* **14** (2021).

17 Xiao, S.-J. *et al.* Traditional Chinese medicine Euodiae Fructus: botany, traditional use, phytochemistry, pharmacology, toxicity and quality control. *Natural Products and Bioprospecting* **13**, 6 (2023).

18 Ibrahim, R. M. *et al.* HPLC-DAD-MS/MS profiling of phenolics from Securigera securidaca flowers and its anti-hyperglycemic and anti-hyperlipidemic activities. *Revista Brasileira de Farmacognosia* **25**, 134-141, doi:10.1016/j.bjp.2015.02.008 (2015).

19 El-sayed, M., Abbas, F. A., Refaat, S., El-Shafae, A. M. & Fikry, E. UPLC-ESI-MS/MS Profile of The Ethyl Acetate Fraction of Aerial Parts of Bougainvillea'Scarlett O'Hara'Cultivated in Egypt. *Egyptian Journal of Chemistry* **64**, 793-806 (2021).

20 Gouveia, S. C. & Castilho, P. C. Characterization of phenolic compounds in Helichrysum melaleucum by high‐performance liquid chromatography with on‐line ultraviolet and mass spectrometry detection. *Rapid Communications in Mass Spectrometry* **24**, 1851-1868 (2010).

21 Fernández-Poyatos, M. d. P., Ruiz-Medina, A., Zengin, G. & Llorent-Martínez, E. J. Phenolic characterization, antioxidant activity, and enzyme inhibitory properties of Berberis thunbergii DC. leaves: A valuable source of phenolic acids. *Molecules* **24**, 4171 (2019).

22 Grati, W. *et al.* HESI-MS/MS Analysis of phenolic compounds from Calendula aegyptiaca fruits extracts and evaluation of their antioxidant activities. *Molecules* **27**, 2314 (2022).

23 Wang, X., Xia, H., Liu, Y., Qiu, F. & Di, X. Simultaneous determination of three glucuronide conjugates of scutellarein in rat plasma by LC–MS/MS for pharmacokinetic study of breviscapine. *Journal of Chromatography B* **965**, 79-84 (2014).

24 Lee, H. G. *et al.* Inhibition of melanogenesis by abietatriene from Vitex trifolia leaf oil. *Natural Product Sciences* **22**, 252-258 (2016).

25 El Sayed, A. M., Ezzat, S. M., El Naggar, M. M. & El Hawary, S. S. In vivo diabetic wound healing effect and HPLC-DAD-ESI-MS/MS profiling of the methanol extracts of eight Aloe species. *Revista Brasileira de Farmacognosia* **26**, 352-362 (2016).

26 Benayad, Z., Gomez-Cordoves, C. & Es-Safi, N. E. Characterization of flavonoid glycosides from fenugreek (Trigonella foenum-graecum) crude seeds by HPLC-DAD-ESI/MS analysis. *Int J Mol Sci* **15**, 20668-20685, doi:10.3390/ijms151120668 (2014).

27 Elbouzidi, A. *et al.* LC–MS/MS Phytochemical Profiling, Antioxidant Activity, and Cytotoxicity of the Ethanolic Extract of Atriplex halimus L. against Breast Cancer Cell Lines: Computational Studies and Experimental Validation. *Pharmaceuticals* **15**, 1156 (2022).

28 Ben Said, R. *et al.* Tentative characterization of polyphenolic compounds in the male flowers of Phoenix dactylifera by liquid chromatography coupled with mass spectrometry and DFT. *International journal of molecular sciences* **18**, 512 (2017).

29 Al-Yousef, H. M. *et al.* UPLC-ESI-MS/MS profile and antioxidant, cytotoxic, antidiabetic, and antiobesity activities of the aqueous extracts of three different Hibiscus Species. *Journal of Chemistry* **2020**, 1-17 (2020).

30 Mitamura, K. & Shimada, K. Derivatization in liquid chromatography/mass spectrometric analysis of neurosteroids. *steroids* **1**, 3-5 (2001).

31 Kobayashi, S., Tokunoha, R., Shibasaki, M., Shinagawa, R. & Murakami-Murofushi, K. Synthesis of 1-O-acylglycerol 2, 3-cyclic phosphate: determination of the absolute structure of PHYLPA, a specific inhibitor of DNA polymerase α. *Tetrahedron letters* **34**, 4047-4050 (1993).

32 Vân Oanh, H. *et al.* CÁC HỢP CHẤT PHENYLPROPANOID GLUCOSIDE TỪ CÂY BẠCH ĐỒNG NỮ CLERODENDRUM PHILIPINUM SCHAUER. *Vietnam Journal of Science and Technology* **48** (2010).

33 Çalis, I., Hosny, M., Yürüker, A., Wright, A. D. & Sticher, O. Inerminosides A and B, two novel complex iridoid glycosides from Clerodendrum inerme. *Journal of natural products* **57**, 494-500 (1994).

34 Zhang, Z. *et al.* LC–MS/MS determination and pharmacokinetic study of seven flavonoids in rat plasma after oral administration of Cirsium japonicum DC. extract. *Journal of ethnopharmacology* **158**, 66-75 (2014).

35 Dais, P. & Boskou, D. Detection and quantification of phenolic compounds in olive oil, olives, and biological fluids. *Olive oil: Minor constituents and health*, 55-107 (2009).

36 Kalo, P., Ollilainen, V., Rocha, J. & Malcata, F. X. Identification of molecular species of simple lipids by normal phase liquid chromatography–positive electrospray tandem mass spectrometry, and application of developed methods in comprehensive analysis of low erucic acid rapeseed oil lipids. *International Journal of Mass Spectrometry* **254**, 106-121 (2006).

37 Hamed, A. I. *et al.* Electrospray ionization mass spectrometry characterization of ubiquitous minor lipids and oligosaccharides in milk of the camel (Camelus dromedarius) and their inhibition of oxidative stress in human plasma. *Journal of dairy science* **103**, 72-86 (2020).

38 Patyra, A., Dudek, M. K. & Kiss, A. K. LC-DAD–ESI-MS/MS and NMR Analysis of Conifer Wood Specialized Metabolites. *Cells* **11**, 3332 (2022).

39 Zheng, X. *et al.* Study on the chemical constituents of Gnaphalium adnatum. *Journal of Yunnan University-Natural Sciences Edition* **37**, 279-284 (2015).
