# Supplementary figures and images for: Phytochemical profiling, metabolomics, and molecular docking studies of Atriplex halimus aerial parts revealing potential insecticidal activity against the malaria vector Anopheles pharoensis
Source: Sci Rep. 2026 May 21;16:15880. doi: 10.1038/s41598-026-52695-1 (PMC13194954; doi:10.1038/s41598-026-52695-1)

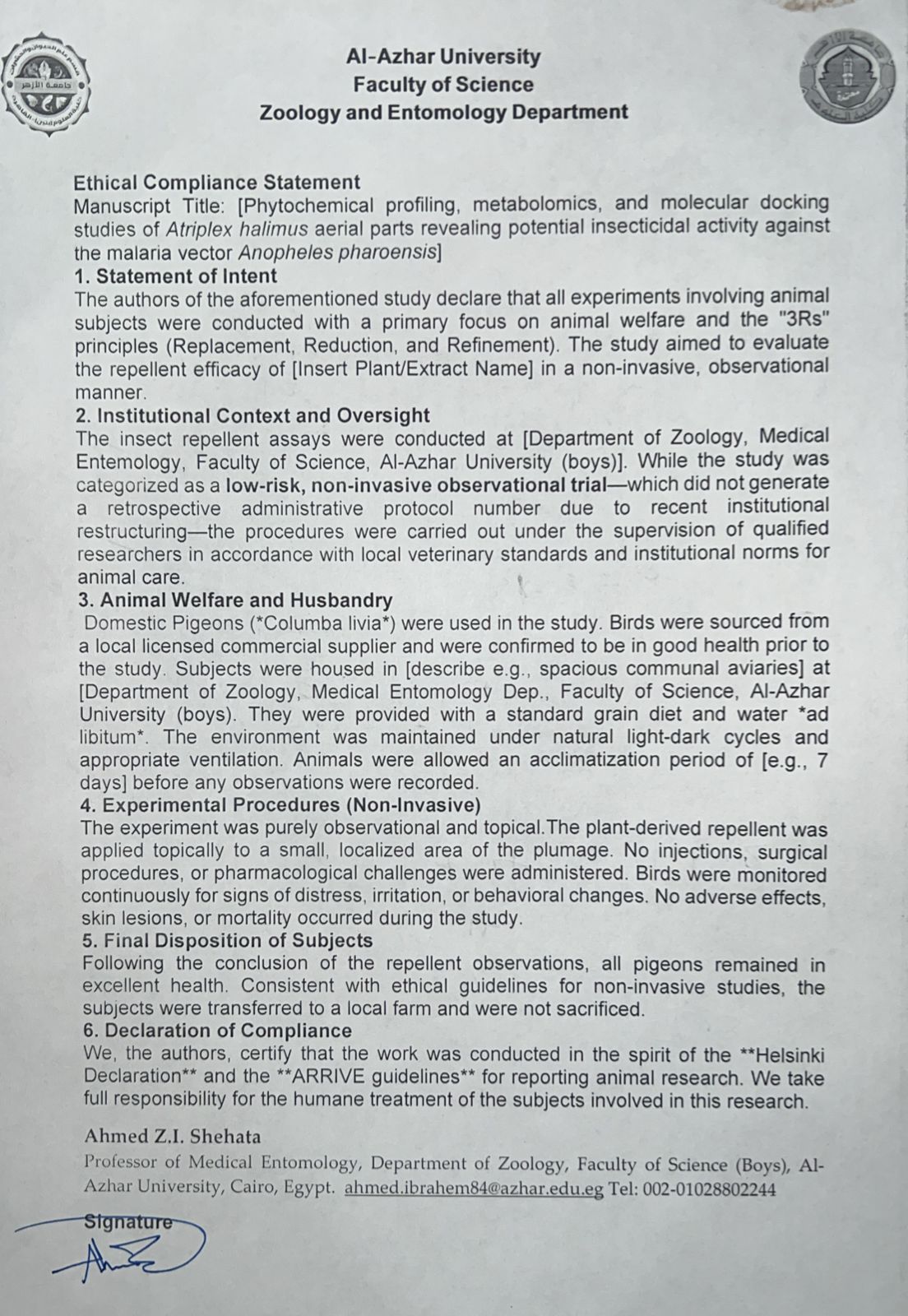

Supplement: Supplementary file 2 — Supplementary Material 2 [file 41598_2026_52695_MOESM2_ESM.jpg]
